# Sulforaphane-Rich Broccoli Sprout Extract Promotes Hair Regrowth in an Androgenetic Alopecia Mouse Model via Enhanced Dihydrotestosterone Metabolism

**DOI:** 10.3390/ijms26157467

**Published:** 2025-08-01

**Authors:** Laxman Subedi, Duc Dat Le, Eunbin Kim, Susmita Phuyal, Arjun Dhwoj Bamjan, Vinhquang Truong, Nam Ah Kim, Jung-Hyun Shim, Jong Bae Seo, Suk-Jung Oh, Mina Lee, Jin Woo Park

**Affiliations:** 1 Department of Biomedicine, Health & Life Convergence Sciences, BK21 Four, Biomedical and Healthcare Research Institute, Mokpo National University, Jeonnam 58554, Republic of Korea; laxmansubedi789@gmail.com (L.S.); dmsqls0749@naver.com (E.K.); sushmitaphuyal54@gmail.com (S.P.); arjun.bamjan@gmail.com (A.D.B.); namahk87@mnu.ac.kr (N.A.K.); s1004jh@gmail.com (J.-H.S.); jbseo@mnu.ac.kr (J.B.S.); 2 Biomedicine Cutting Edge Formulation Technology Center, Mokpo National University, Jeonnam 58554, Republic of Korea; 3 College of Pharmacy and Research Institute of Life and Pharmaceutical Sciences, Sunchon National University, Suncheon, Jeonnam 57922, Republic of Korea; ddle@scnu.ac.kr (D.D.L.); quangvtruong00@gmail.com (V.T.); 4 Department of Natural Cosmetics Science and Smart Beautytech Research Institute, Sunchon National University, Suncheon, Jeonnam 57922, Republic of Korea; 5 College of Pharmacy and Natural Medicine Research Institute, Mokpo National University, Jeonnam 58554, Republic of Korea; 6 Research & Development, Ecoworld Pharm Co. Ltd., Jeonnam 57304, Republic of Korea; sj.oh@ecoworldpharm.com

**Keywords:** broccoli sprout extract, sulforaphane, androgenetic alopecia, hair regeneration, dihydrotestosterone, hepatic DHT metabolism

## Abstract

Androgenetic alopecia (AGA) is a common progressive hair loss disorder driven by elevated dihydrotestosterone (DHT) levels, leading to follicular miniaturization. This study investigated sulforaphane-rich broccoli sprout extract (BSE) as a potential oral therapy for AGA. BSE exhibited dose-dependent proliferative and migratory effects on keratinocytes, dermal fibroblasts, and dermal papilla cells, showing greater in vitro activity than sulforaphane (SFN) and minoxidil under the tested conditions, while maintaining low cytotoxicity. In a testosterone-induced AGA mouse model, oral BSE significantly accelerated hair regrowth, with 20 mg/kg achieving 99% recovery by day 15, alongside increased follicle length, density, and hair weight. Mechanistically, BSE upregulated hepatic and dermal DHT-metabolizing enzymes (Akr1c21, Dhrs9) and activated Wnt/β-catenin signaling in the skin, suggesting dual actions via androgen metabolism modulation and follicular regeneration. Pharmacokinetic analysis revealed prolonged SFN plasma exposure following BSE administration, and in silico docking showed strong binding affinities of key BSE constituents to Akr1c2 and β-catenin. No systemic toxicity was observed in liver histology. These findings indicate that BSE may serve as a safe, effective, and multitargeted natural therapy for AGA. Further clinical studies are needed to validate its efficacy in human populations.

## 1. Introduction

Hair loss is a multifactorial condition that affects individuals across a broad spectrum of age groups and demographic backgrounds [1]. Beyond its physical presentation, hair holds substantial psychosocial importance, serving as a symbol of personal identity, vitality, and social engagement [2]. As a result, hair loss can lead to significant psychological distress, diminished self-esteem, and a reduced quality of life [3]. Among the various types of alopecia, androgenetic alopecia is the most common, particularly in men. Androgenetic alopecia (AGA) is characterized by the progressive miniaturization of hair follicles, which leads to the transformation of thick, pigmented terminal hairs into thin, non-pigmented vellus hairs, predominantly affecting the frontotemporal and vertex regions of the scalp [4,5]. This process is driven by a disrupted hair growth cycle, marked by a shortened anagen (growth) phase and a prolonged telogen (resting) phase [6].

The pathogenesis of AGA involves a complex interplay of genetic predisposition, hormonal imbalance, and local biochemical changes within the follicular microenvironment. A central factor in this process is dihydrotestosterone (DHT), a potent androgen synthesized from testosterone via the action of 5α-reductase enzymes [7]. Elevated DHT levels act on androgen receptors in dermal papilla cells, downregulating the transcription of growth-promoting genes while upregulating inhibitors such as transforming growth factor-β and dickkopf-1. This molecular signaling cascade accelerates the premature transition from the anagen (growth) phase to the catagen (regression) phase, leading to suppressed keratinocyte proliferation, follicular regression, and, ultimately, hair thinning and loss [4,8].

Current therapeutic options for AGA remain limited to finasteride and minoxidil, the only food drug administration (FDA)-approved treatments. However, minoxidil primarily accelerates hair follicle growth without effectively preventing further hair loss in AGA. Additionally, its formulation often contains excipients such as propylene glycol, which can cause greasiness, itching, and contact dermatitis. The use of marketed 5% minoxidil is frequently associated with adverse effects such as dermatitis and headaches, contributing to poor treatment compliance. Finasteride, on the other hand, inhibits 5α-reductase, thus reducing the conversion of testosterone to DHT. However, its efficacy may be limited in certain cases, particularly when 5β-reductase is locally upregulated and alters steroid hormone metabolism, potentially contributing to region-specific DHT generation from testosterone, or when DHT is regenerated via back-conversion under hyperandrogenic conditions. Notably, even low concentrations of DHT can exert potent biological effects due to its high affinity for androgen receptors, thereby sustaining AGA pathogenesis [9,10,11,12]. Moreover, finasteride is associated with well-documented adverse effects, including sexual dysfunction and mood disturbances, and requires continuous long-term administration to maintain efficacy [13,14]. These limitations highlight the urgent need for safer and more effective therapies that can target DHT with greater specificity. In addition, improving patient adherence through more convenient and better-tolerated treatment options is essential for achieving sustained clinical outcomes. In this context, phytochemicals and naturally derived compounds have attracted growing interest due to their favorable safety profiles, high tolerability, and ability to modulate multiple biological pathways [15,16].

A promising therapeutic strategy involves enhancing the catabolism of DHT rather than solely inhibiting its synthesis. This metabolic pathway is primarily mediated by hepatic hydroxysteroid dehydrogenases (HSDs), particularly 3α-HSDs, which convert DHT into inactive metabolites such as 3β,17β-androstanediol in the presence of NADPH [17]. Unlike direct enzyme inhibition, this approach reduces circulating DHT levels without disrupting the broader androgen biosynthetic pathway, thereby potentially minimizing systemic adverse effects.

Sulforaphane (SFN), a dietary isothiocyanate abundantly found in cruciferous vegetables such as broccoli, is well recognized for its potent antioxidant, anti-inflammatory, and hormone-regulatory properties [18]. SFN activates the nuclear factor erythroid 2-related factor 2/antioxidant response element (Nrf2/ARE) pathway, a key regulator of cellular defense against oxidative stress, and upregulates critical DHT-metabolizing enzymes, including Akr1c1, Akr1c2, and Dhrs9 [19,20]. These mechanistic actions position SFN as a promising therapeutic candidate for AGA. However, the clinical application of SFN is significantly limited by its poor chemical stability, low aqueous solubility (~1.85 mg/mL in water at 25 °C), and short biological half-life (approximately 2.2 h in humans). SFN is highly susceptible to degradation when exposed to oxidants, light, and heat during storage [21]. After 5 weeks, the residual SFN content remained at 96.56 ± 0.15%, 95.18 ± 0.20%, and 78.45 ± 0.28% when stored at −20 °C, 4 °C, and 26 °C, respectively [22]. Moreover, SFN is unstable under acidic conditions; at pH 1.2, which mimics gastric fluid, nearly 90% degrades within 1 h, severely limiting intestinal absorption [23]. Its biological half-life is also short, ranging from 1 to 2 h, which may be insufficient for the sustained suppression of DHT in AGA treatment [24]. Collectively, these limitations compromise SFN’s therapeutic efficacy and reduce patient adherence [25,26].

To overcome the limitations of pure SFN, we employed a broccoli sprout extract (BSE) enriched with naturally stabilized SFN and other bioactive phytochemicals. In this matrix, SFN is thought to be protected by surrounding natural components that may shield it from light, moisture-induced degradation, and gastric acid, thereby enhancing its chemical stability and oral bioavailability [22,23,27]. Our previous studies demonstrated that BSE, prepared using an optimized endogenous extraction protocol, exhibits markedly improved chemical stability, enhanced antioxidant and anti-inflammatory activities, and comparable oral bioavailability to that of synthetic or isolated SFN [28,29]. Despite its complex composition, BSE achieved pharmacokinetic parameters equivalent to pure SFN, including similar peak plasma concentrations and absorption kinetics [29]. These findings suggest that the natural matrix components present in BSE do not impede, but may in fact enhance, the systemic delivery of SFN. Moreover, BSE exhibited significantly greater antioxidant activity, with oxygen radical absorbance capacity (ORAC) values nearly four times higher than those of pure SFN. This enhanced activity likely results from the presence of other antioxidant compounds such as polyphenols, flavonoids, terpenoids, and ascorbic acid, which are known to contribute strongly to the fluorescein–AAPH-based ORAC response [29]. Taken together, these advantages position BSE as a promising natural formulation that delivers the pharmacological benefits of SFN in a more stable, bioavailable, and physiologically active form.

This study evaluated the therapeutic potential of BSE in the context of AGA. In particular, we quantified the SFN content in BSE and investigated its in vitro effects on cell proliferation and wound healing in keratinocytes (HaCaT), dermal fibroblasts (CCD-986sk), and human dermal papilla (HDP) cells. We also conducted pharmacokinetic analyses in rats and assessed the in vivo efficacy of BSE in a testosterone-induced AGA mouse model. Furthermore, molecular investigations examined the expression of hepatic and dermal DHT-metabolizing enzymes, as well as the activation of signaling pathways associated with hair growth. Additionally, chemical investigation of BSE demonstrated the key components which have beneficial effects on the targets through in silico approach. Collectively, our findings offer mechanistic insights and provide a strong therapeutic rationale for the development of BSE as a novel, orally administered treatment for AGA.

## 2. Results

### 2.1. BSE Characterization

#### 2.1.1. BSE Chemical Profile

**Figure 1:**
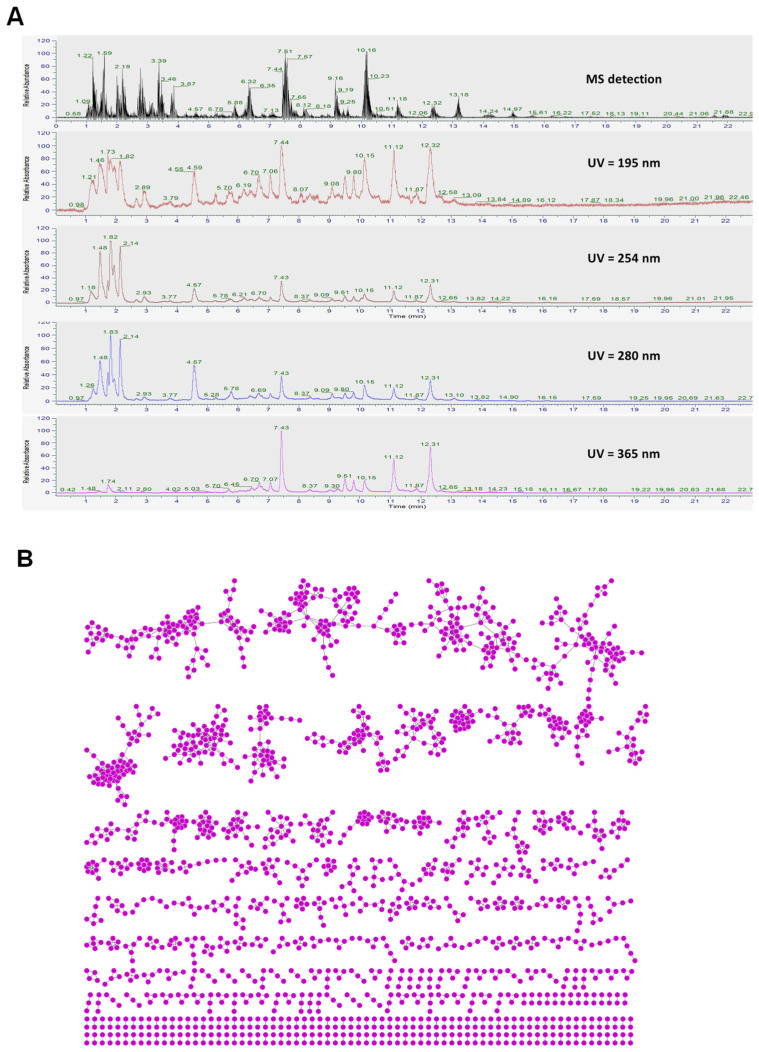

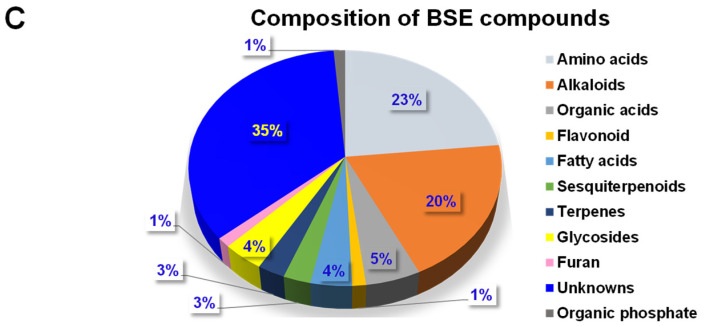
(**A**) Chemical profile of broccoli sprout extract (BSE) detected using mass spectrometry (MS) and multiple ultraviolet (UV) wavelengths. (**B**) Feature-based molecular networking (FBMN) of BSE visualized using Cytoscape 3.10.1. (**C**) Chemical classification of compounds identified in BSE.

The comprehensive profiles of BSE were obtained using both chromatographic and spectrometric techniques, combining MS with multiple ultraviolet (UV) detection wavelengths (195, 254, 280, and 365 nm) (Figure 1). The MS trace revealed a diverse range of compounds with prominent peaks, supporting the presence of major constituents. UV detection at 195 nm showed high intensity for peaks corresponding to compounds with little or no UV absorbance at higher wavelengths. Conversely, major peaks were observed at 254 nm, 280 nm, and especially at 365 nm (Figure 1A), indicating the presence of flavonoids and conjugated phenolic derivatives [30]. These chemical fingerprints underscore the diversity and complexity of BSE, justifying the use of molecular networking for compound annotation and classification.

#### 2.1.2. Feature-Based Molecular Networking (FBMN) Analysis

FBMN is a powerful computational and analytical approach used to organize, visualize, and interpret complex mass spectrometry data, especially in nontargeted metabolomics in the context of scientific research [31,32]. FBMN integrates both structural (MS/MS) and chromatographic (LC retention time) information, enabling researchers to distinguish between closely related compounds such as positional isomers and stereoisomers that might otherwise be indistinguishable using mass spectrometry alone. This method significantly enhances the efficiency of data analysis and the discovery of novel metabolites or natural products [31,32]. Using untargeted liquid chromatography–tandem mass spectrometry (LC-MS/MS) analysis combined with UV and isotopic mass patterns, 77 compound structures were annotated (Table S1). The MS/MS-based molecular network (Figure 1B) involves an untargeted fragmentation analysis of all compounds in the extract, aligning MS/MS spectra and assembling them into nodes based on spectral similarity to reference spectra from the global natural products social molecular networking (GNPS) web platform and other public spectral libraries (Table S2). Among the identified compounds, peak 60 (L-sulforaphane) was identified at level 1 by comparing it with the reference standard (Figure S1). Additionally, other compounds were identified at level 2 [33], with confidence values within the range of 758–1000. Peaks 1 (arginine, *m*/*z* 175.1186, retention at 1.216 min), 2 (L-proline, *m*/*z* 116.0703, retention at 1.307 min), 6 (valine, *m*/*z* 118.0859, retention at 1.490 min), 9 (adenine, *m*/*z* 136.0615, retention at 1.589 min), 16 (adenosine, *m*/*z* 268.1030, retention at 2.004 min), 17 ([6,10a-dihydroxy-4-(hydroxymethyl)-4,7,11b-trimethyl-9-oxo-1,2,3,4a,5,6,6a,7,11,11a-decahydronaphtho[2,1-f][1]benzofuran-5-yl] acetate, *m*/*z* 431.2033, retention at 2.054 min), 18 (norleucine, *m*/*z* 132.1014, retention at 2.195 min), 24 (phenylalanine, *m*/*z* 166.0859, retention at 3.808 min), 57 (silodosin, *m*/*z* 496.2430, retention at 9.158 min), and 60 (L-sulforaphane, *m*/*z* 178.0351, retention at 10.164 min) were estimated as major components based on their peak intensities in the extract chromatogram (Figure S2).

#### 2.1.3. Chemical Composition and Classification

Based on spectral matching and chemical classification (Figure 1C), BSE was found to consist predominantly of amino acids (23%), followed by alkaloids (20%), organic acids (5%), fatty acids, glycosides (each 4%), terpenes, sesquiterpenoids (each 3%), and unclassified compounds. Minor classes included organic phosphates, furans, and flavonoids. The rich chemical diversity revealed through both chromatographic profiling and molecular networking highlights BSE as a promising source of bioactive natural products.

### 2.2. In Vitro Cell Proliferation Assay

**Figure 2:**
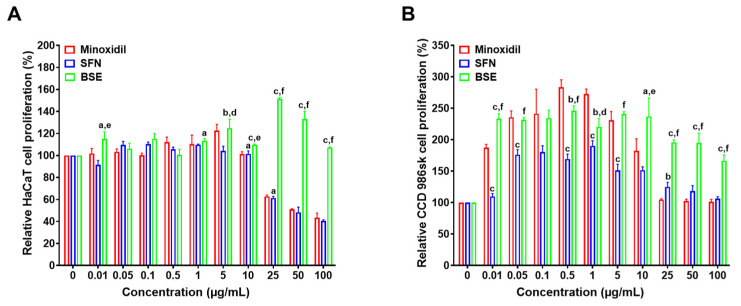

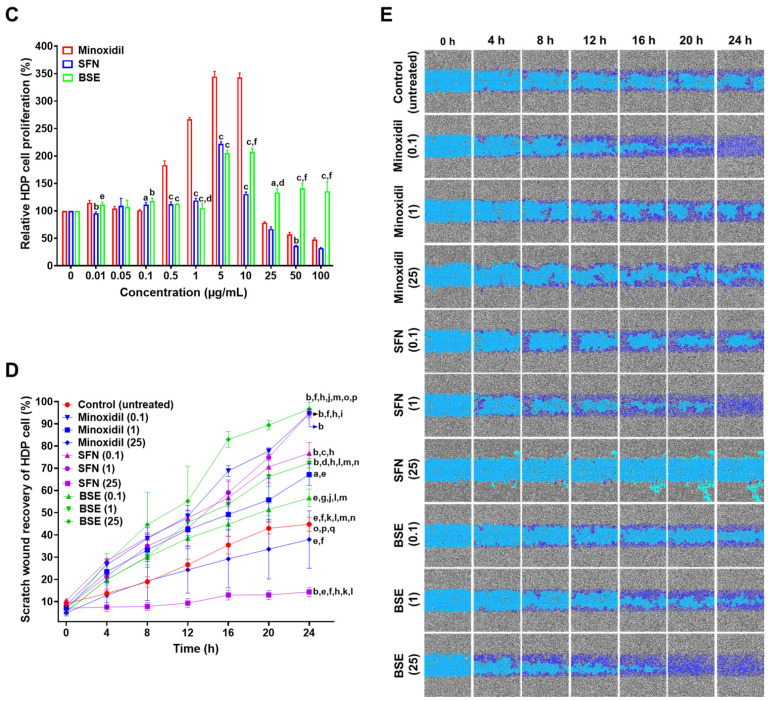
In vitro cell proliferation assay. Relative cell proliferation of (**A**) HaCaT, (**B**) CCD 986sk, and (**C**) HDP cells after treatment with minoxidil, SFN, and BSE at various concentrations. ^a^ *p* < 0.05, ^b^ *p* < 0.01, and ^c^ *p* < 0.001 compared to minoxidil; ^d^ *p* < 0.05, ^e^ *p* < 0.01, and ^f^ *p* < 0.001 compared to SFN. Values are presented as mean ± SD (*n* = 3 for each group). (**D**) Time course curve of relative scratch wound recovery of HDP cells after incubation with various concentrations of minoxidil, SFN, and BSE. ^a^ *p* < 0.01 and ^b^ *p* < 0.001 compared to the control; ^c^ *p* < 0.05, ^d^ *p* < 0.01, and ^e^ *p* < 0.001 compared to minoxidil (0.1); ^f^ *p* < 0.001 compared to minoxidil (1); ^g^ *p* < 0.01 and ^h^ *p* < 0.001 compared to minoxidil (25); ^i^ *p* < 0.05, ^j^ *p* < 0.01, and ^k^ *p* < 0.001 compared to SFN (0.1); ^l^ *p* < 0.001 compared to SFN (1); ^m^ *p* < 0.001 compared to SFN (25); ^n^ *p* < 0.05 and ^o^ *p* < 0.001 compared to BSE (0.1); ^p^ *p* < 0.001 compared to BSE (1); ^q^ *p* < compared to BSE (25). Values are presented as mean ± SD (*n* = 3 for each group). (**E**) Representative microscope images of scratch wounds in HDP cells. Color code: gray—initial cell state; light sky blue—scratch wound area; bluish purple—wound recovery area.

The effects of BSE, SFN, and minoxidil on cell proliferation were evaluated in HaCaT, CCD-986sk, and HDP cells (Figure 2A–C). In HaCaT cells, all three treatments showed minimal proliferative activity at low concentrations (0.01–1 µg/mL) (Figure 2A). However, BSE induced a marked increase in proliferation at higher concentrations (25–100 µg/mL), with the most pronounced effect observed at 25 µg/mL, resulting in a 59% increase relative to the untreated control (Figure 2A). Conversely, minoxidil and SFN exhibited cytotoxic effects at concentrations above 10 µg/mL, as indicated by decreased cell viability.

In CCD-986sk cells, both BSE and minoxidil significantly enhanced proliferation at lower concentrations (0.01–0.1 µg/mL) (Figure 2B). Notably, minoxidil treatment induced increases of 184% and 173% at concentrations of 0.5 and 1 µg/mL, respectively, surpassing the effects seen with BSE. However, BSE exhibited more stable and sustained proliferative effects across a broader concentration range. Compared to minoxidil, BSE treatment resulted in 17.6%, 66.0%, 91.3%, 92.4%, and 65.7% higher cell proliferation at 5, 10, 25, 50, and 100 µg/mL, respectively (Figure 2B). Both minoxidil (≥10 µg/mL) and SFN (≥5 µg/mL) decreased proliferation in CCD-986sk cells, indicating dose-dependent cytotoxicity.

In HDP cells, minoxidil elicited a concentration-dependent proliferative response, peaking at 344% at 1 µg/mL, followed by a marked decline at concentrations above 10 µg/mL (Figure 2C). SFN showed moderate stimulatory effects, with proliferation reaching approximately 200% between 0.5 and 5 µg/mL, but decreasing at higher doses. Conversely, BSE induced a stable, concentration-dependent increase in proliferation, maintaining consistent activity up to 1 µg/mL and reaching 205% and 207% at 5 and 10 µg/mL, respectively (Figure 2C). Notably, BSE sustained proliferative activity at higher concentrations where both minoxidil and SFN exhibited reduced efficacy.

Taken together, these findings suggest that BSE exerts superior and concentration-stable proliferative effects across all tested hair-related cell lines, including HaCaT, CCD-986sk, and HDP cells. Conversely, minoxidil and SFN demonstrated strong activity only within specific concentration ranges and were limited by cytotoxicity at higher doses.

### 2.3. In Vitro Scratch Wound Assay

To further evaluate the effects of BSE on cellular activities relevant to hair regeneration, an in vitro scratch wound recovery assay was conducted using HDP cells over 24 h. Cells were treated with minoxidil, SFN, or BSE at concentrations of 0.1, 1, and 25 µg/mL and compared to an untreated control (Figure 2D,E). The control group showed minimal wound closure, with only 35.4% closure at 16 h, reflecting the baseline migratory capacity of HDP cells. Among the minoxidil-treated groups, 0.1 µg/mL induced the highest wound closure at this time point (68.8%), whereas 1 and 25 µg/mL treatments resulted in lower recovery rates (49.2% and 29.2%, respectively), indicating a concentration-dependent decline in efficacy. Notably, this contrasts with the proliferation assay, where minoxidil at 1 µg/mL demonstrated the strongest proliferative effect, highlighting a disconnect between proliferation and migratory responses. SFN showed optimal wound recovery at 1 µg/mL (71.5% closure at 16 h), whereas the highest concentration (25 µg/mL) caused significant cell detachment and reduced recovery, indicating cytotoxicity. Conversely, BSE treatment produced dose-dependent improvements in wound closure, achieving 83.0% closure at 16 h with 25 µg/mL, outperforming all other treatments—including minoxidil at its optimal concentration—by 14.7%. This trend persisted at 20 h, with BSE (25 µg/mL) showing the greatest recovery rate (89.4%), followed by SFN (1 µg/mL, 74.9%) and minoxidil (0.1 µg/mL, 77.7%). SFN at 25 µg/mL consistently showed impaired recovery, remaining below the control, confirming its concentration-dependent cytotoxicity in HDP cells. At the final 24 h time point, all three optimal concentrations produced strong wound closure: minoxidil (0.1 µg/mL) reached 94.7%, SFN (1 µg/mL) reached 94.8%, and BSE (25 µg/mL) demonstrated the highest efficacy with 96.7% closure.

To further validate the pro-migratory effects of BSE, a Boyden chamber assay was conducted using HDP cells. The results demonstrated that minoxidil, SFN, and BSE significantly enhanced HDP cell migration compared to the untreated control in a dose-dependent manner, with distinct efficacy profiles for each compound (Figure S3). The untreated control was set at a baseline migration of 100%. Minoxidil at 0.1 µg/mL increased migration to 157%, but higher concentrations showed reduced effects (136% at 1 µg/mL and 80.0% at 25 µg/mL). SFN also promoted migration at lower doses (141% at 0.1 µg/mL and 150% at 1 µg/mL), but migration significantly declined at 25 µg/mL (16.7%), suggesting cytotoxicity at higher concentrations. In contrast, BSE exhibited a consistent and dose-dependent enhancement, reaching 125% at 0.1 µg/mL, 146% at 1 µg/mL, and peaking at 165% at 25 µg/mL. Notably, BSE at 25 µg/mL demonstrated the highest migration-promoting effect among all treatments, indicating its strong pro-migratory potential in HDP cells.

Collectively, these findings indicate that while minoxidil and SFN exhibit strong wound-healing effects only within narrow concentration ranges, BSE offers a broader, dose-dependent, and cytocompatible effect, supporting its potential as a natural alternative for enhancing dermal papilla cell migration and promoting hair follicle regeneration.

### 2.4. In Vivo Oral Absorption in Rats

**Figure 3:**
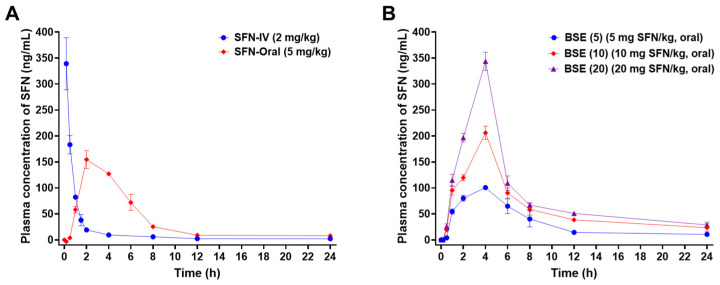
In vivo pharmacokinetic study in rats. Venous plasma concentration of SFN following a single (**A**) IV injection (SFN-IV, 2 mg/kg) and oral administration (SFN-Oral, 5 mg/kg). (**B**) Plasma concentration of SFN after oral administration of BSE (5) (5 mg/kg equivalent to SFN), BSE (10) (10 mg/kg equivalent to SFN), and BSE (20) (20 mg/kg equivalent to SFN). Values are presented as mean ± SD (*n* = 3 for each group).

The plasma concentration–time profiles of SFN were evaluated following the oral administration of pure SFN (SFN-Oral, 5 mg/kg) and BSE containing SFN at equivalent doses of 5, 10, and 20 mg/kg (Figure 3, Table 1). These were compared to an intravenous (IV) reference group (SFN-IV, 2 mg/kg) to estimate oral bioavailability.

The oral administration of SFN (5 mg/kg) showed a delayed time to reach the maximum plasma concentration (T_max_; 2 h) and a reduced maximum plasma concentration (C_max_; 155 ± 17 ng/mL) compared to the IV group but demonstrated enhanced systemic exposure with an area under the plasma concentration–time curve between zero and the last measurable plasma concentration (AUC_last_) of 874 ± 46 ng·h/mL, corresponding to an oral bioavailability of 87 ± 5% (Figure 3A, Table 1). When BSE was administered at an equivalent SFN dose (5 mg/kg), the C_max_ decreased further to 101 ± 3 ng/mL, whereas AUC_last_ remained comparable at 794 ± 73.2 ng·h/mL, yielding a similar oral bioavailability of 79 ± 7% (Figure 3, Table 1). The prolonged plasma retention of SFN in the BSE group, likely due to a slower elimination half-life, may explain this comparable systemic exposure despite the lower peak concentrations.

Interestingly, the dose escalation of BSE to 10 and 20 mg/kg resulted in a decline in relative oral bioavailability (73 ± 1% and 52 ± 1%, respectively), suggesting a nonlinear absorption profile, possibly due to the saturation of transporter-mediated uptake or increased first-pass metabolism. Nonetheless, compared to the reference dose of oral BSE (5 mg/kg equivalent to SFN), the 10 and 20 mg/kg doses of BSE exhibited marked dose-dependent increases in C_max_ by approximately 104% and 241%, respectively. These increases were accompanied by a prolonged half-life (14 and 13 h), resulting in significantly enhanced AUC_last_ values—86% and 162% higher than that of the 5 mg/kg dose (Figure 3, Table 1).

Notably, BSE at 20 mg/kg achieved a C_max_ (344 ± 17 ng/mL) comparable to that of SFN-IV, indicating that the high oral doses of BSE can deliver therapeutically relevant systemic SFN concentrations. These results collectively suggest that BSE provides the sustained systemic exposure of SFN with dose-dependent pharmacokinetics, although absorption efficiency may decrease at higher doses.

Thus, BSE may serve as a promising oral delivery system for SFN, especially for chronic conditions such as AGA, where sustained systemic exposure and the modulation of DHT are therapeutically advantageous.

### 2.5. In Vivo Hair Regrowth Efficacy

Hair regrowth dynamics were evaluated in a testosterone-induced AGA mouse model following the oral administration of BSE at doses of 5, 10, and 20 mg/kg, alongside standard treatments with 1% topical minoxidil and oral finasteride. Hair cycle progression was monitored by changes in dorsal skin pigmentation: the appearance of black pigmentation indicated anagen phase initiation (active hair growth), whereas pink pigmentation signified the maintenance of the telogen phase (resting state). Assessments were conducted at six time points: days 0, 3, 6, 9, 12, and 15 following treatments.

**Figure 4:**
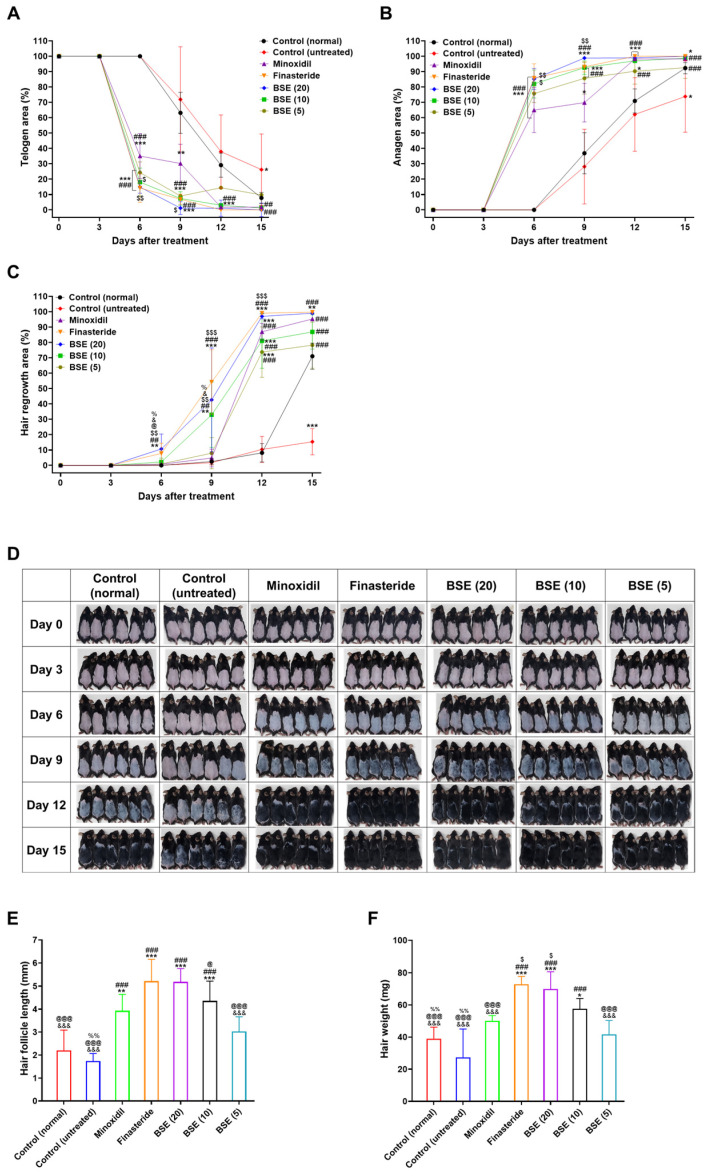

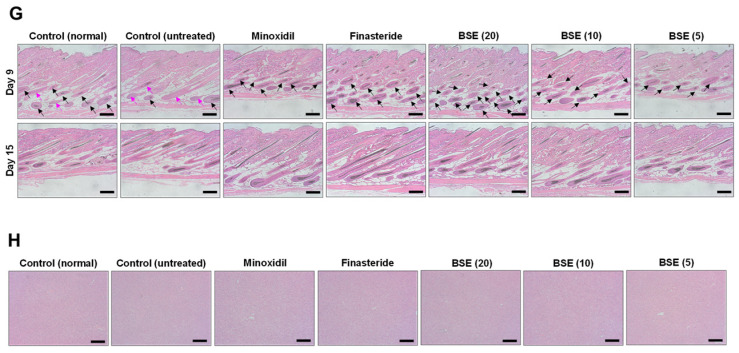
In vivo evaluation of hair regrowth efficacy in the following groups: control (normal) (non-androgenic model, no treatment), control (untreated) (androgenic model, no treatment), minoxidil (androgenic model, 1% minoxidil, topical application, once daily), finasteride (androgenic model, 1 mg/kg finasteride, oral administration, once daily), and broccoli sprout extract (BSE 20, 10, or 5) (androgenic model, BSE administered orally at doses of 20, 10, or 5 mg/kg, once daily). (**A**) Time course curve showing reduction in telogen phase (pink area). (**B**) Time course curve showing an increase in the anagen phase (black area), representing the transition from the telogen phase to the anagen phase on dorsal skin. (**C**) Time course curve showing hair regrowth on dorsal skin. * *p* < 0.05, ** *p* < 0.01, and *** *p* < 0.001 compared to control (normal); ^##^ *p* < 0.01 and ^###^ *p* < 0.001 compared to control (untreated); ^$^ *p* < 0.05, ^$$^ *p* < 0.01, and ^$$$^ *p* < 0.001 compared to minoxidil; ^@^ *p* < 0.05 compared to finasteride; ^&^ *p* < 0.05 compared to BSE (20); ^%^ *p* < 0.05 compared to BSE (10). Values are presented as mean ± SD (*n* = 6 for each group). (**D**) Representative photographs of mouse dorsal skin showing hair regrowth. (**E**) Length of hair follicles at 15 days after treatment. (**F**) Hair weight for each group on day 15 after treatment. * *p* < 0.05 and ** *p* < 0.01 compared to control (normal); ^###^ *p* < 0.001 compared to control (untreated); ^$^ *p* < 0.05 compared to minoxidil; ^@^ *p* < 0.05 and ^@@@^ *p* < 0.001 compared to finasteride; ^&&&^ *p* < 0.001 compared to BSE (20); ^%%^ *p* < 0.01 compared to BSE (10). Values are presented as mean ± SD (*n* = 6 for each group). (**G**) Hematoxylin and eosin (H&E) staining of mouse skin sections on days 9 and 15 post-treatment to observe hair regrowth trends. Scale bar = 100 µm. Pink arrow—telogen phase and black arrow—anagen phase. (**H**) Cross-sectional images of liver tissue stained with H&E to assess toxicity. Scale bar = 100 µm.

During the initial treatment phase (days 0 and 3), all experimental groups exhibited a uniform hair cycle status, with 100% telogen area, 0% anagen area, and no visible hair regrowth, confirming effective synchronization across groups (Figure 4A–D). In the untreated AGA control group (testosterone-induced but without any subsequent treatment), hair cycle progression remained markedly suppressed throughout the study period, characterized by persistent telogen phase dominance and minimal hair regrowth—the hallmarks of androgen-driven alopecia. Conversely, AGA mice treated with minoxidil, finasteride, or BSE (5, 10, or 20 mg/kg; equivalent to BSE) exhibited accelerated hair cycle activation, evidenced by earlier transitions into the anagen phase and more pronounced hair regrowth compared to both untreated AGA controls (testosterone-treated mice without subsequent intervention) and normal controls (non-AGA mice without any hair growth treatment). No significant differences in pigmentation or hair regrowth were observed among the treatment groups by day 3. However, by day 6, distinct telogen-to-anagen transitions became apparent, especially in the finasteride and BSE (20) groups, indicating the onset of therapeutic efficacy (Figure 4A,B).

On day 6, the minoxidil and finasteride groups showed substantial telogen-to-anagen conversion, with 65.0% and 85.8% of telogen areas transitioning, respectively, compared to the untreated control (Figure 4A,B). BSE treatments also demonstrated dose-dependent improvements, with telogen-to-anagen conversions of 85.2%, 82.0%, and 75.7% for BSE (20), BSE (10), and BSE (5), respectively. The enhanced conversion observed with minoxidil is likely attributable to its vasodilatory effects and the indirect stimulation of follicular activity, whereas the earlier onset of skin darkening seen with finasteride and BSE is related to their DHT-modulating effects. In terms of hair regrowth, minoxidil and finasteride groups showed 0.7% and 7.76% coverage of the telogen area, respectively. Among the BSE groups, BSE (20) exhibited optimal hair regrowth, covering 10.7% of the telogen area at day 6—representing increases of 9.97%, 2.91%, 8.45%, and 9.77% compared to minoxidil, finasteride, BSE (10), and BSE (5), respectively (Figure 4C,D).

By day 9, a clear divergence in treatment efficacy emerged. Both the finasteride and BSE (20) groups exhibited near-complete telogen-to-anagen transitions, with telogen areas reduced to 6.94% and 1.17%, respectively, with the corresponding anagen areas reaching 93.1% and 98.4%. Hair regrowth also advanced significantly, with oral finasteride achieving 54.2% and oral BSE (20) reaching 42.6% (Figure 4C). Conversely, minoxidil-treated mice showed a 69.8% anagen area but only 4.79% hair regrowth, suggesting a delay between anagen induction and visible hair fiber production. Moderate improvements were observed in the BSE (10) and BSE (5) groups, with hair regrowth rates of 32.9% and 7.80%, respectively (Figure 4A–D).

By day 12, BSE (20) showed a substantial increase in hair regrowth, rising from 42.6% to 97.0%, representing a 54.4% improvement compared to day 9 and achieving hair regrowth comparable to finasteride. BSE (10) also demonstrated hair regrowth similar to that of the minoxidil treatment by day 12 (Figure 4C,D).

By the end of the treatment period (day 15), the highest dose of BSE (20 mg/kg) achieved 99.0% hair regrowth, matching the efficacy of finasteride (99.0%) and surpassing minoxidil (95.0%) and the untreated control (52.2%) (Figure 4C,D). BSE (10) and BSE (5) also showed substantial improvements, reaching 86.9% and 73.5% hair regrowth, respectively. Collectively, these results indicate that BSE—particularly at the 20 mg/kg dose, which has higher C_max_ and AUC_last_ despite lower oral bioavailability compared to the 10 and 5 mg/kg doses—exerts a strong dose-dependent effect in promoting hair follicle cycling and regrowth, achieving efficacy comparable to finasteride and being superior to minoxidil in the AGA model.

### 2.6. Hair Follicle Length and Hair Weight

Hair follicle length was assessed on day 15 by measuring randomly plucked newly grown hairs from the dorsal skin of each mouse. As shown in Figure 4E, follicle length in the untreated control group was 1.27-fold shorter than that of the normal control group, confirming the suppressive effect of androgen on hair growth. Treatment with minoxidil (1%) and finasteride (1 mg/kg, oral) led to 2.24- and 3.00-fold increases in hair follicle length compared to the untreated group, respectively. Similarly, BSE treatment produced a dose-dependent increase in follicle length, with 2.99-, 2.51-, and 1.74-fold increases observed in the 20, 10, and 5 mg/kg BSE groups, respectively. Compared to minoxidil, BSE at 20 and 10 mg/kg induced 1.32- and 1.11-fold longer follicles, respectively (Figure 4E). Notably, BSE (20 mg/kg) showed comparable efficacy to finasteride in promoting hair follicle elongation.

At the end of the treatment, newly regrown dorsal hair was harvested and weighed to assess the overall mass of hair regrowth. Hair weight in the untreated control group was significantly lower than that of the normal control, reflecting delayed or impaired hair development under androgenic influence (Figure 4F). Treatment with minoxidil and finasteride increased hair weight by 82.5% and 165%, respectively, compared to the untreated group. BSE also demonstrated a dose-dependent enhancement in hair regrowth, with BSE (20) resulting in hair weight increases comparable to those observed with minoxidil and finasteride in the mouse model. Notably, BSE (20) resulted in a 39.8% increase in hair weight relative to minoxidil and showed hair weights comparable to those observed in the finasteride-treated group (Figure 4F), suggesting that BSE may promote not only follicular elongation but also the development of denser and more robust hair shafts.

To further evaluate the effect of BSE on hair follicle density and morphological phase progression, H&E staining of dorsal skin sections was performed at multiple time points. By day 9, histological differences among the treatment groups became evident. The untreated group showed reduced follicle numbers and the delayed development of follicle bulbs compared to both the normal control and treated groups (Figure 4G). Minoxidil-treated mice exhibited more advanced bulb formation than either control group, whereas the follicle number and development were most pronounced in the finasteride and BSE (20) groups. Additionally, a clear dose–response relationship was observed within the BSE-treated groups. By day 15, both the finasteride and BSE (20) groups demonstrated well-developed follicles entering or maintaining the anagen phase, further supporting the potential use of BSE as an alternative treatment for AGA (Figure 4G).

Importantly, no physical abnormalities were observed in any mice throughout the 15-day treatment period with oral BSE or finasteride. H&E staining of liver tissue revealed no histological signs of toxicity in any group, including those treated with BSE at doses of 20, 10, or 5 mg/kg, indicating the safety of repeated oral administration of BSE for AGA treatment (Figure 4H).

### 2.7. Plasma Testosterone and DHT Levels

**Figure 5:**
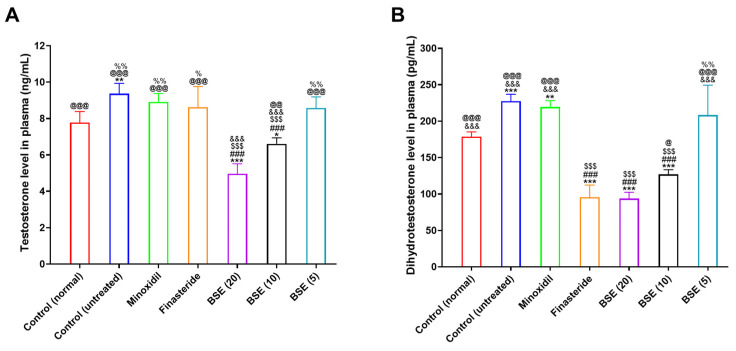
Plasma level of androgenic hormone related to hair growth. In vivo plasma levels of (**A**) testosterone and (**B**) dihydrotestosterone in control (normal) (non-androgenic model, no treatments), control (untreated) (androgenic model, no treatments), minoxidil (androgenic model, minoxidil (1%), topical application, once in a day), finasteride (androgenic model, finasteride 1 mg/kg, oral administration, once in a day), and broccoli sprout extract (BSE) (BSE 20, 10, or 5) (androgenic model, BSE with its equivalent dose of 20, 10, or 5 mg/kg, oral administration, once in a day). * *p* < 0.05, ** *p* < 0.01, and *** *p* < 0.001 compared to control (normal); ^###^ *p* < 0.001 compared to control (untreated); ^$$$^ *p* < 0.001 compared to minoxidil; ^&&&^ *p* < 0.001 compared to finasteride; ^@^ *p* < 0.05, ^@@^ *p* < 0.01, and ^@@@^ *p* < 0.001 compared to BSE (20); ^%^ *p* < 0.05 and ^%%^ *p* < 0.01 compared to BSE (10). Values are presented as mean ± SD (*n* = 6 for each group).

The effects of BSE on plasma testosterone and DHT levels were evaluated and compared with those in minoxidil, finasteride, and control groups (Figure 5). In the untreated alopecia control group (AGA mice treated with testosterone), both testosterone and DHT levels were elevated compared to the normal control group (non-AGA mice without testosterone treatment), confirming the successful induction of AGA via the daily subcutaneous administration of testosterone (5 mg/kg) for five consecutive days (Figure 5A).

Finasteride treatment did not significantly reduce plasma testosterone levels, consistent with its known mechanism of selectively inhibiting the conversion of testosterone to DHT without affecting testosterone production. Similarly, minoxidil showed no detectable effect on either testosterone or DHT levels, which aligns with its role as a vasodilator without direct involvement in androgen metabolism.

Conversely, BSE exhibited a dose-dependent suppressive effect on plasma testosterone levels. Although the 5 mg/kg dose had no significant impact, BSE at 10 mg/kg and 20 mg/kg resulted in 1.42- and 1.88-fold lower testosterone levels, respectively, compared to the untreated control group (Figure 5A). This reduction suggests that BSE may influence upstream regulatory mechanisms beyond DHT conversion.

Regarding DHT levels, finasteride treatment led to a marked 2.38-fold reduction compared to the untreated control, consistent with its inhibition of 5α-reductase, the enzyme responsible for converting testosterone to DHT (Figure 5B). Notably, BSE at 10 and 20 mg/kg also significantly decreased plasma DHT by 1.79- and 2.43-fold, respectively. The DHT-lowering effect observed in the BSE (20) group was comparable to that of finasteride, indicating that BSE may possess multiple mechanisms of action, such as activating DHT-degrading enzymes in the liver, a potent inhibitory effect on 5α-reductase activity, and lower plasma testosterone levels via various unknown mechanisms.

These findings suggest that BSE exerts dual anti-androgenic effects by dose-dependently reducing both testosterone and DHT levels. Unlike minoxidil, which acts peripherally through vasodilation, BSE appears to target the hormonal axis involved in hair loss, supporting its potential as a natural therapeutic agent for managing AGA.

### 2.8. Restoration of DHT Metabolism and Wnt Signaling by BSE Treatment

In AGA, excessive DHT production suppresses hair growth and promotes hair follicle miniaturization. Conversely, the enhanced degradation of DHT through the upregulation of 3α-HSD enzymes—such as aldo-keto reductase family member Akr1c21 and dehydrogenase/reductase SDR family member Dhrs9—may help mitigate the progression of hair loss. To investigate whether BSE influences the hepatic expression of these enzymes, we performed Western blot analysis on liver tissues from testosterone-induced AGA model mice.

**Figure 6:**
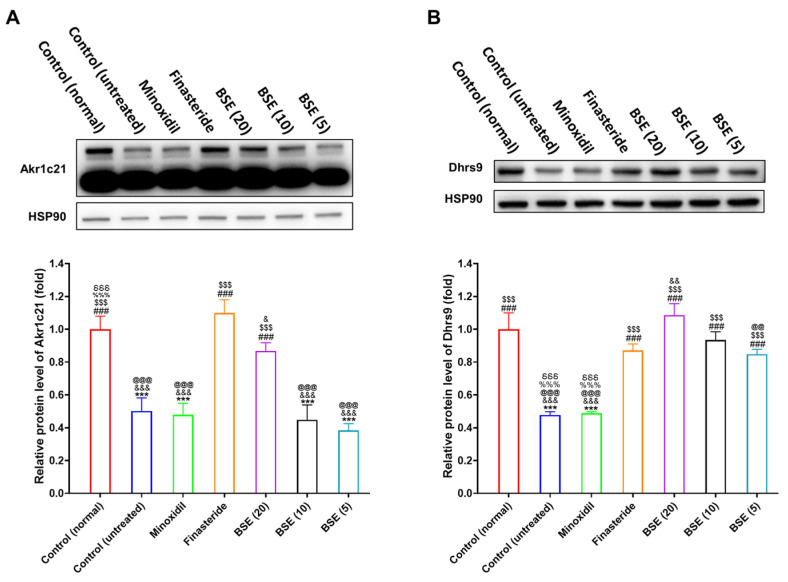
In vivo activation of 3α-HSD enzymes in the liver 15 days post-treatment. Relative protein expression levels of (**A**) Akr1c21 and (**B**) Dhrs9 in liver tissues were quantified from Western blot band intensities. Bar graphs represent quantification of band intensities normalized to HSP90. *** *p* < 0.001 compared to control (normal) (non-androgenic model, no treatments); ^###^ *p* < 0.001 compared to control (untreated) (androgenic model, no treatments); ^$$$^ *p* < 0.001 compared to minoxidil (androgenic model, minoxidil (1%), topical application, once in a day); ^&^ *p* < 0.05, ^&&^ *p* < 0.01, and ^&&&^ *p* < 0.001 compared to finasteride (androgenic model, finasteride 1 mg/kg, oral administration, once in a day); ^@@^ *p* < 0.01 and ^@@@^ *p* < 0.001 compared to BSE (20) (androgenic model, BSE with its equivalent dose of 20 mg/kg, oral administration, once in a day); ^%%%^ *p* < 0.001 compared to BSE (10) (androgenic model, BSE with its equivalent dose of 10 mg/kg, oral administration, once in a day); ^δδδ^ *p* < 0.001 compared to BSE (5) (androgenic model, BSE with its equivalent dose of 5 mg/kg, oral administration, once in a day). Values are presented as mean ± SD (*n* = 3 for each group).

In the AGA control group [control (untreated)], testosterone administration significantly suppressed the hepatic expression of both Akr1c21 and Dhrs9, indicating impaired DHT catabolism associated with the disease (Figure 6, Figures S4 and S5). Among the treatment groups, minoxidil showed no significant effect on the expression of either enzyme. Conversely, finasteride markedly upregulated both Akr1c21 and Dhrs9, consistent with its established role in reducing DHT levels via 5α-reductase inhibition.

BSE administration also increased the expression of 3α-HSD enzymes in a dose-dependent manner. Notably, Akr1c21 was significantly upregulated only at the highest BSE dose (20 mg/kg, SFN equivalent), whereas Dhrs9 expression increased consistently across all BSE-treated groups (5, 10, and 20 mg/kg) (Figure 6). These findings suggest that BSE, likely through its SFN content, enhances the hepatic expression of key enzymes involved in DHT metabolism.

Collectively, these findings suggest that BSE not only inhibits DHT formation but also promotes its degradation by upregulating 3α-HSD enzymes. This dual modulatory effect on androgen metabolism underscores BSE’s potential as a natural therapeutic candidate for the prevention and treatment of AGA.

The transition of hair follicles from the telogen (resting) phase to the anagen (growth) phase is tightly regulated by interactions between mesenchymal and epithelial cells and critically depends on the activation of the Wnt/β-catenin signaling pathway. To assess whether BSE influences this pathway in a testosterone-induced AGA mouse model, we analyzed the expression of β-catenin and lymphoid enhancer-binding factor 1 (Lef-1) in dorsal skin tissues using Western blot.

**Figure 7:**
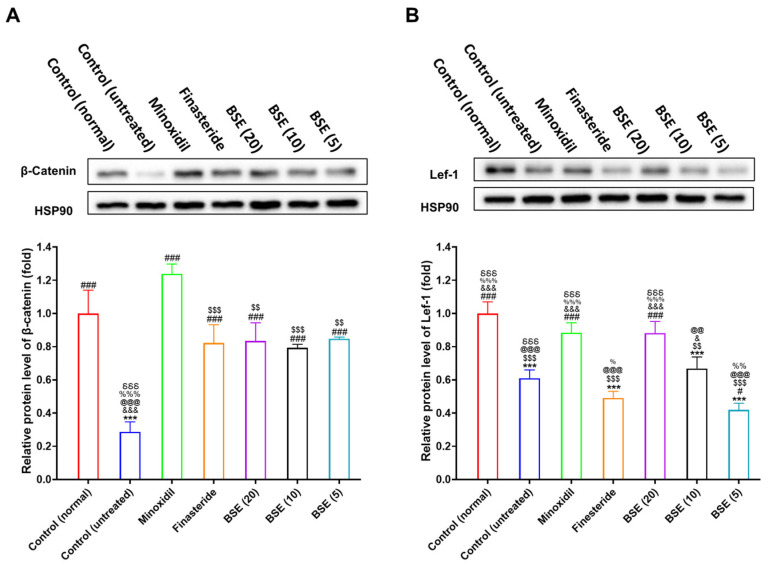
In vivo β-catenin levels in the dorsal skin of mice at 15 days post-treatment. Relative protein levels of (**A**) β-catenin and (**B**) Lef-1 in the dorsal skin of mice were quantified from Western blot band intensities. Bar graphs represent quantification of band intensities normalized to HSP90. *** *p* < 0.001 compared to control (normal) (non-androgenic model, no treatments); ^#^ *p* < 0.05 and ^###^ *p* < 0.001 compared to control (untreated) (androgenic model, no treatments); ^$$^ *p* < 0.01 and ^$$$^ *p* < 0.001 compared to minoxidil (androgenic model, minoxidil (1%), topical application, once in a day); ^&^ *p* < 0.05 and ^&&&^ *p* < 0.001 compared to finasteride (androgenic model, finasteride 1 mg/kg, oral administration, once in a day); ^@@^ *p* < 0.01 and ^@@@^ *p* < 0.001 compared to BSE (20) (androgenic model, BSE with its equivalent dose of 20 mg/kg, oral administration, once in a day); ^%^ *p* < 0.05, ^%%^ *p* < 0.01, and ^%%%^ *p* < 0.001 compared to BSE (10) (androgenic model, BSE with its equivalent dose of 10 mg/kg, oral administration, once in a day); ^δδδ^ *p* < 0.001 compared to BSE (5) (androgenic model, BSE with its equivalent dose of 5 mg/kg, oral administration, once in a day). Values are presented as mean ± SD (*n* = 3 for each group).

In the testosterone-induced AGA model group, both β-catenin and Lef-1 expression levels were significantly downregulated compared to the normal control group, indicating the suppression of Wnt signaling due to androgen exposure (Figure 7 and Figure S6). Treatment with topical minoxidil or oral finasteride significantly restored β-catenin expression. Although Lef-1 expression showed a positive trend in the minoxidil-treated group, no such trend was observed in the finasteride-treated group, and neither increase reached statistical significance.

BSE treatment at doses of 5, 10, and 20 mg/kg led to a dose-dependent restoration of β-catenin expression. The β-catenin levels observed in the BSE-treated groups were comparable to those in the clinically established treatment groups—namely, 1% topical minoxidil and 1 mg/kg oral finasteride—indicating the reactivation of Wnt signaling (Figure 7A). Notably, Lef-1 expression was significantly upregulated only in the high-dose BSE group (20 mg/kg), further supporting BSE’s potential to modulate the downstream targets of the Wnt pathway (Figure 7B).

These results indicate that BSE activates the Wnt/β-catenin signaling cascade, likely via its bioactive component SFN, thereby facilitating the re-entry of hair follicles into anagen. This mechanistic evidence supports the therapeutic potential of BSE as a natural agent for treating or preventing AGA.

### 2.9. Molecular Docking Approach

**Figure 8:**
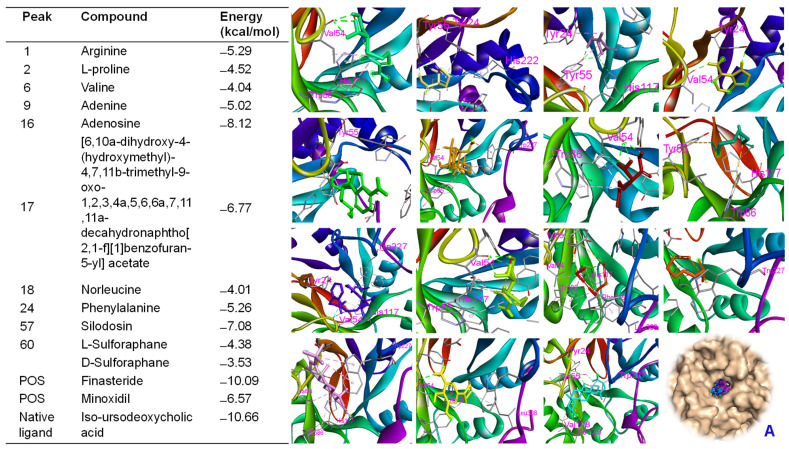
Binding affinities and interactions of major compounds—arginine (light green), L-proline (light yellow), valine (dark pink), adenine (limon), adenosine (green), [6,10a-dihydroxy-4-(hydroxymethyl)-4,7,11b-trimethyl-9-oxo-1,2,3,4a,5,6,6a,7,11,11a-decahydronaphtho[2,1-f][1]benzofuran-5-yl] acetate (light orange), norleucine (dark red), phenylalanine (dark green), silodosin (violet), L-sulforaphane (waxy green), D-sulforaphane (red), finasteride (magenta), minoxidil (yellow), and the native ligand (iso-ursodeoxycholic acid, marine)—with amino acid residues when docked into the Ark1c2 protein (PDB ID: 1IHI). A: All compounds were docked into the active region of the protein.

**Figure 9:**
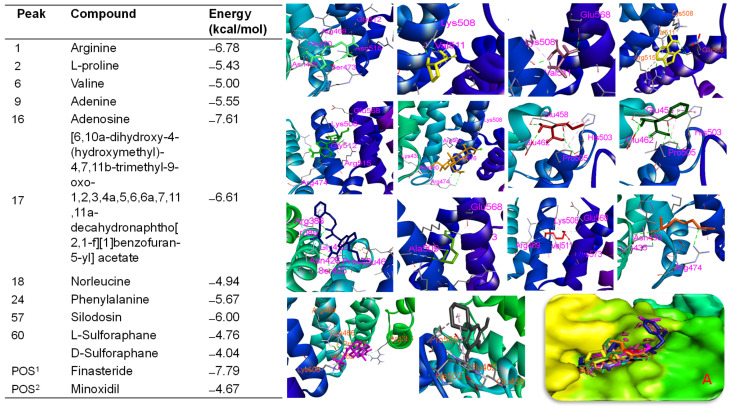
Binding affinities and interactions of major compounds—arginine (light green), L-proline (light yellow), valine (dark pink), adenine (yellow), adenosine (green), [6,10a-dihydroxy-4-(hydroxymethyl)-4,7,11b-trimethyl-9-oxo-1,2,3,4a,5,6,6a,7,11,11a-decahydronaphtho[2,1-f][1]benzofuran-5-yl] acetate (light orange), norleucine (dark red), phenylalanine (dark green), silodosin (violet), L-sulforaphane (waxy green), D-sulforaphane (orange), finasteride (magenta), and minoxidil (black)—with amino acid residues when docked into the β-catenin protein (PDB ID: 1JDH). A: All compounds were docked into the active region of the protein.

BSE demonstrated biological effects targeting hair growth in both in vitro and in vivo assays. Therefore, in silico studies were conducted to verify the bioactivities of BSE by assessing the binding affinities of its major peaks toward proteins involved in hair growth activation. Molecular docking analysis indicated that all major peaks occupied the same binding site as the native ligand (iso-ursodeoxycholic acid) and the two positive controls (finasteride and minoxidil) on the Ark1c2 protein [34]. These ligands were docked into the binding pocket of the Ark1c2 protein. Notably, peaks 16, 17, and 57 showed docking scores of ΔG = −8.12 kcal/mol, −6.77 kcal/mol, and −7.08 kcal/mol, respectively, which were lower than that of minoxidil (ΔG = −6.57 kcal/mol) under the same in silico conditions. Peaks 16, 17, and 57 may interact with key amino acid residues such as VAL54, TYR55, VAL128, ILE129, and TRP227 (Figure S7)—residues identified as crucial in the binding pose of the protein [34]. Similarly, the native ligand and the two positive controls also interacted with these residues through hydrogen bonding or hydrophobic interactions (Figure 8). Additionally, these major peaks were evaluated for their binding affinities toward β-catenin [34]. The results showed that peak 16 exhibited a significant binding affinity of −7.61 kcal/mol, compared to pharmaceutical control, finasteride (ΔG = −7.79 kcal/mol). Additionally, arginine (ΔG = −6.78 kcal/mol), adenine (ΔG = −5.55 kcal/mol), and L-proline (ΔG = −5.43 kcal/mol) each showed specific hydrogen bonding interactions, especially involving LYS508 and GLU568—residues frequently observed as contact points across multiple ligands (Figure 9). These findings align with previous studies that emphasize the critical role of these residues in ligand recognition and β-catenin function. Our data revealed that several major peaks exhibited stronger binding affinities than L-SFN, which may explain the superior bioactivity of BSE compared to SFN in both in vitro and in vivo studies.

Conversely, SFN derivatives (L-SFN and D-SFN) and isothiocyanato compounds exhibited moderate binding affinities (ΔG: −4.76 to −4.04 kcal/mol) and primarily engaged in hydrophobic interactions, particularly with TYR306, PRO493, and ASP549. The pharmaceutical reference minoxidil showed a comparatively lower binding energy (ΔG = −4.67 kcal/mol), serving as a control that highlights the stronger binding profiles of other compounds. Key residues involved in ligand–receptor interactions were identified, i.e., LYS508, GLU568, ALA547, PRO493, and TYR306. These residues likely constitute a conserved interaction hotspot on β-catenin, making them critical targets for ligand anchoring.

The major components of BSE exhibited significant binding interactions with both β-catenin and Ark1c2 proteins in silico. Docking studies revealed that these components interacted with key residues within the active sites of the proteins, resulting in low binding energy scores. Our previous report suggested that BSE displayed a high content of SFN (461 ± 10 ppm) [29]. However, BSE’s interfering hair growth activity may be significantly influenced by the activity of other components. These computational findings align with the observed differences in biological activity between SFN- and BSE-treated samples, suggesting a potential mechanism for hair growth activation.

### 2.10. Molecular Dynamic Simulation Analysis of Ligand–Protein Complexes

**Figure 10:**
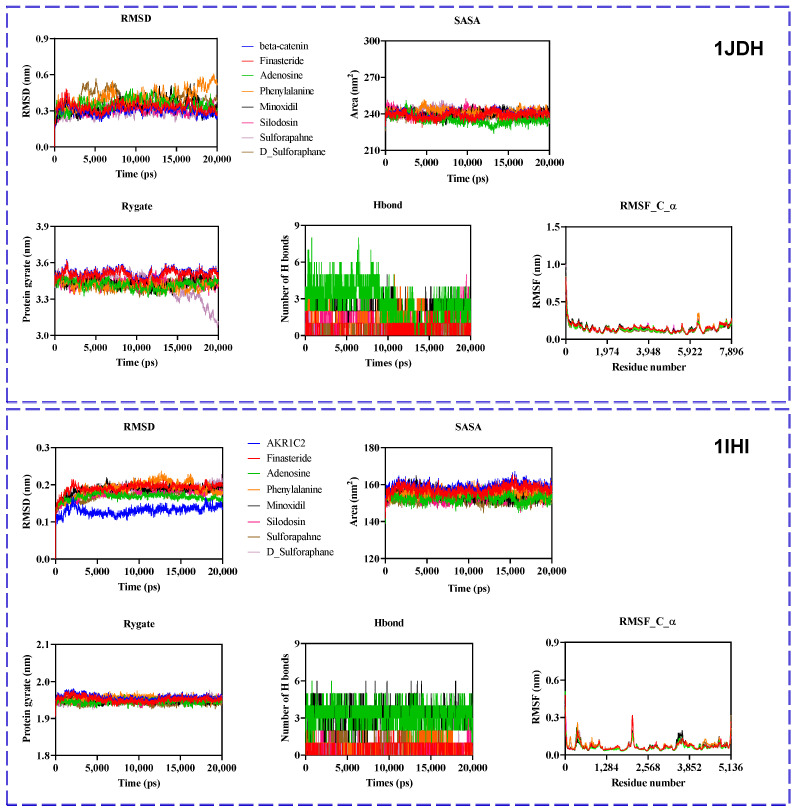
Molecular dynamic simulation analyses of protein–ligand complexes for 1JDH: β-catenin and transcription factor 4 (Tcf4) (top panel); 1IHI: AKR1C2 with NADP^+^ and ursodeoxycholate (bottom panel). Each panel corresponds to a target protein, with multiple ligands—including finasteride, adenosine, phenylalanine, minoxidil, silodosin, 1a46_trimethyl, L-sulforaphane, and D-sulforaphane—assessed for their interaction profiles during the simulation.

The β-catenin and Akr1c2 proteins were used to assess dynamic parameters, including stability, compactness, and interaction profiles of the selected compounds with their target proteins over the simulation timeframe. Conformational behavior and contact stability were evaluated throughout the simulation using various metrics, such as the root mean square deviation (RMSD), root mean square fluctuation (RMSF), solvent-accessible surface area [20], number of hydrogen bonds (H-bonds), and radius of gyration (Rg) (Figure 10).

#### 2.10.1. β-Catenin (1JDH) Complexes

##### RMSD and Structural Stability

The RMSD profiles of the β-catenin complexes indicated moderate fluctuations, generally stabilizing within the range of 0.25–0.45 nm. Notably, complexes with finasteride and SFN or other phytoconstituents present in BSE exhibited consistently low RMSD values, suggesting high conformational stability and minimal structural deviation throughout the simulation.

##### Rg and Compactness

Rg values remained relatively stable across all complexes, fluctuating around 3.3–3.4 nm. This suggests that ligand binding did not cause significant global structural expansion or contraction of the β-catenin protein.

##### Solvent Accessibility and Flexibility

SASA values ranged between 240 and 270 nm^2^, showing only minor variations among the ligands. These stable SASA profiles indicate consistent surface exposure throughout the simulation, supporting the stable folding and compactness of the protein–ligand complexes.

##### H-Bond

Hydrogen bonding analysis revealed diverse interaction profiles. Ligands such as finasteride and SFN analogs formed a greater number of persistent hydrogen bonds, reinforcing their potential for high-affinity binding.

##### RMSF and Residue-Level Flexibility

The RMSF plots highlight minimal fluctuations across the protein backbone, with slight increases observed near terminal residues and loop regions (Figure 10). This indicates local stability in the ligand-binding regions, particularly for D-SFN and adenosine, which maintained interactions characterized by low RMSF variability.

#### 2.10.2. Akr1c2 (1IHI) Complexes

##### RMSD and Stability

Akr1c2 complexes exhibited RMSD values below 0.3 nm for most ligands, indicating excellent stability. Complexes with adenosine, finasteride, and SFN showed the most stable trajectories, with minimal deviation throughout the simulation.

##### Rg and Structural Compactness

Rg values remained within the range of 2.3–2.4 nm for all complexes, demonstrating that ligand binding did not significantly affect the compactness of Akr1c2.

##### SASA

SASA values across Akr1c2 complexes consistently ranged between 160 and 170 nm^2^, further confirming structural stability. Finasteride and SFN derivatives exhibited the lowest SASA fluctuations.

##### Hydrogen Bonding

Akr1c2–ligand systems exhibited more diverse hydrogen bonding dynamics compared to the β-catenin system. Notably, D-SFN and finasteride maintained a higher number of hydrogen bonds throughout the simulation, reinforcing their strong binding potential.

##### RMSF

RMSF analysis revealed low residue fluctuations throughout the protein, with modest peaks observed at flexible loop regions. Ligands such as adenosine, minoxidil, and finasteride, which cause minimal fluctuations, suggest a strong stabilizing effect on binding.

Overall, fluctuations were observed for the apo protein (without ligand) and for the complexes (protein + ligands) for each target protein. The MD result suggested that the established complexes showed slightly higher MD parameters compared to the apo protein. However, the RMSF value showed a minor change, meaning that our complexes are stable and capable of effective binding.

## 3. Discussion

This study demonstrates that SFN-rich BSE exerts therapeutic effects against AGA through multiple mechanisms, including endocrine modulation, follicular regeneration, and molecular signaling pathway modulation. For this purpose, the oral administration of BSE was selected as the primary route to evaluate systemic hair regrowth efficacy, considering its potential to modulate hepatic DHT synthesis and maintain sufficient plasma SFN exposure. Although topical application may offer localized effects at the hair follicle level, the complex composition of BSE and its limited solubility in conventional topical vehicles present formulation challenges. Additionally, the risk of residual accumulation on the skin surface may hinder its efficacy. Therefore, future studies may focus on developing skin-penetrating formulations to explore the topical utility of BSE in the treatment of androgenic alopecia. Thus, oral delivery was assumed to be the most practical and effective route for BSE at present.

One of the key findings of this study is the dose-dependent reduction in plasma DHT and testosterone levels following oral BSE administration. Unlike finasteride, which selectively inhibits 5α-reductase and blocks the conversion of testosterone to DHT without affecting upstream androgen synthesis, BSE appears to influence both DHT formation and circulating testosterone levels. Although the upstream regulators of androgen biosynthesis were not directly examined, the observed decrease in testosterone may reflect an indirect modulation of androgen homeostasis, potentially mediated by hepatic enzyme activity.

In support of this, the hepatic upregulation of key 3α-hydroxysteroid dehydrogenases (Akr1c21 and Dhrs9) in BSE-treated animals suggests the enhanced peripheral catabolism of DHT, leading to reduced systemic androgen levels. However, we acknowledge that critical upstream enzymes involved in steroidogenesis—such as HSD3B1/2 and CYP17A1—were not evaluated in this study. This limits our ability to determine whether BSE directly affects testosterone biosynthesis or whether the observed effects result from enhanced peripheral metabolism or systemic endocrine feedback mechanisms.

Additionally, this study did not assess the local expression of 3α-HSD isozymes within hair follicles, particularly in dermal papilla cells, which are central to androgen-mediated hair regulation. This omission limits conclusions regarding the follicle-specific modulation of androgen metabolism by BSE. Future studies employing techniques such as immunohistochemistry, laser-capture microdissection, or single-cell transcriptomics will be necessary to further elucidate the site-specific and mechanistic actions of BSE in androgen metabolism.

In addition to its systemic hormonal effects, BSE showed strong local efficacy in promoting hair regrowth. Histological and quantitative analyses confirmed improvements in hair follicle length, density, and maturation, with the 20 mg/kg dose producing outcomes comparable to finasteride or minoxidil. These results suggest that BSE not only preserves but actively regenerates hair follicle architecture in the presence of androgenic stress.

Mechanistically, these effects appear to be linked to the activation of the Wnt/β-catenin pathway. Western blot results showed dose-dependent increases in total β-catenin and Lef-1 expression in BSE-treated skin tissues. However, since total skin lysates were used, these data do not differentiate between cytoplasmic and nuclear β-catenin pools. Given that β-catenin must localize to the nucleus to exert transcriptional activity, our findings cannot confirm the transcriptional activation of Wnt signaling. Future work employing nuclear–cytoplasmic fractionation or immunofluorescence microscopy will be essential to verify β-catenin nuclear translocation.

Furthermore, we did not examine downstream Wnt target gene expression (e.g., *Axin2, Cyclin D1*) at the mRNA level, which would distinguish whether the observed increases in protein levels result from transcriptional activation or post-translational stabilization [34,35]. This distinction remains important and warrants further investigation. Previous studies, including Han et al. (2017), have shown that SFN—the key bioactive component of BSE—upregulates β-catenin and Cyclin D1 in neural stem cells, supporting the plausibility of transcriptional Wnt activation by SFN in our model as well [36]. Nevertheless, tissue-specific responsiveness to SFN should be carefully considered, and follow-up studies will clarify its activity in skin and follicular compartments.

The efficacy of BSE was further supported by in vitro cell proliferation and wound healing assays, which demonstrated the broad concentration-dependent enhancement of keratinocyte and fibroblast activity. Unlike minoxidil, which exhibited narrow concentration-dependent efficacy and cytotoxicity at higher doses, BSE maintained stable and non-toxic proliferation-promoting effects across a wider range. This suggests a more robust and safer cellular response, enhancing its potential for long-term therapeutic use.

Pharmacokinetic analysis demonstrated that the oral administration of BSE led to the sustained plasma exposure of SFN, with a prolonged half-life and dose-dependent increases in AUC. Although oral bioavailability decreased at higher doses, systemic SFN levels remained within a therapeutically relevant range—particularly at 20 mg/kg, where the C_max_ was comparable to that of IV administration. These findings suggest that BSE serves as an effective oral delivery matrix for SFN, potentially protecting it from acid-mediated degradation in gastric conditions (pH 1.2) and enabling intestinal absorption [23]. In addition to SFN, other BSE constituents may also be absorbed based on their solubility and partitioning characteristics along the gastrointestinal tract. For instance, compounds such as arginine and phenylalanine are known to support hair regrowth through distinct mechanisms. Arginine, a precursor of nitric oxide, enhances scalp microcirculation and stimulates follicular activity, thereby reducing hair thinning and promoting new hair growth. Phenylalanine contributes to protein synthesis essential for keratin formation, improves nutrient uptake in hair follicles, and helps restore the hydrophobic barrier of the hair shaft, supporting strength and growth [37,38,39]. Although the current study did not evaluate the pharmacokinetics of these non-SFN constituents due to experimental limitations, their potential contribution to the overall efficacy of BSE cannot be excluded. Further studies are warranted to quantify their concentrations in BSE, assess their bioavailability, and elucidate their individual or synergistic roles in promoting hair regeneration.

In silico molecular docking and dynamic simulation studies supported the pharmacological findings, revealing strong binding affinities between major BSE constituents and target proteins involved in DHT metabolism and Wnt signaling. Interacting with both protein targets, finasteride, adenosine, and SFN derivatives consistently demonstrated favorable interaction profiles. Their low RMSD values, stable Rg, minimal SASA fluctuations, and strong hydrogen bonding suggest robust and stable binding. The molecular dynamics simulations provide supportive evidence suggesting that these compounds, particularly finasteride, adenosine, and SFN analogs, maintain stable interactions with both β-catenin and Akr1c2. These findings suggest that the selected compounds may serve as promising multifunctional bioactive candidates for the development of hair growth activators. Compounds such as peaks 16, 17, and 57 exhibited strong binding affinity toward proteins targeted to promote hair growth, suggesting a plausible molecular basis for the extract’s observed in vivo efficacy.

Collectively, these results support the potential of BSE as a promising natural therapeutic agent for the treatment of AGA. Its unique ability to modulate both systemic and local androgen pathways, restore follicular signaling, and provide sustained SFN exposure may offer advantages over conventional therapies. Based on the observed efficacy at 20 mg/kg in ~25 g mice, the estimated human equivalent dose (HED) for a 60 kg adult ranges from approximately 9.2 mg (safety-adjusted starting dose) to 91.98 mg/day [40]. In addition, previous studies have shown that raw broccoli and broccoli sprouts can enhance SFN bioavailability. However, the absorption of specific constituents from BSE may vary depending on the geographic origin and cultivation conditions of the broccoli sprouts. Therefore, accurate dose estimation requires quantitative analysis of the active chemical components in the extract. Furthermore, the food matrix and meal composition can significantly influence the absorption and bioavailability of BSE [28]. Taken together, further clinical investigations are warranted to evaluate the efficacy, safety, and optimal dosing regimen of oral BSE supplementation in humans, particularly for its potential use in the long-term management of androgen-mediated hair loss.

Lastly, this study has general limitations. The structural differences between mouse and human hair follicles may affect translational relevance. The relatively short duration (2 weeks) may not capture the long-term regenerative effects of BSE. Finally, the complex interplay of other signaling pathways beyond DHT metabolism and Wnt/β-catenin was not explored here. Future studies should expand the scope of this study to fully elucidate the multifaceted mechanisms underlying BSE-mediated hair regrowth.

## 4. Materials and Methods

### 4.1. Materials

SFN was obtained from TargetMol Chemicals Inc. (Catalog. TQ0207, 99.79% purity, Medford, MA, USA). Phenethyl isothiocyanate (PHE), used as the internal standard (IS) for SFN, along with testosterone and sodium carboxymethyl cellulose (NaCMC), was purchased from Sigma-Aldrich Inc. (St. Louis, MO, USA). Acetonitrile, methanol, water, and formic acid used for mobile phase preparation were procured from Waters Corp. (Milford, MA, USA). All chemicals were of analytical grade, and all solvents were of LC-MS/MS grade.

### 4.2. Animals

Male Sprague–Dawley rats (6 weeks old, approximately 250 g) and male C57BL/6J mice (6 weeks old, approximately 20 g) were obtained from G-Bio (Gwangju, Republic of Korea). Animals were maintained under standard laboratory conditions, including a temperature of 25 ± 2 °C, a relative humidity of 55 ± 10%, and a 12 h light/dark cycle. The animals had ad libitum access to a standard rodent diet (PicoLab^®^ Rodent Diet 20, Cat. No. 5053; 20% protein, 4.5% fat; Nestlé Purina PetCare, St. Louis, MO, USA) and ion-sterilized tap water. All experimental procedures were conducted in accordance with the National Institutes of Health Guidelines for the Care and Use of Laboratory Animals and were approved by the Institutional Animal Care and Use Committee (IACUC) of Mokpo National University (Jeonnam, Approval Nos. MNU-IACUC-2024-003, approved on 16 May 2024; and MNU-IACUC-2024-005, approved on 29 August 2024).

### 4.3. BSE Preparation and Quantification

#### 4.3.1. Sample Preparation and Analysis

The BSE sample was obtained by following our previous report without modification [29], with additional details provided here to ensure reproducibility. Fresh broccoli sprouts were thoroughly rinsed with deionized water to remove surface impurities while preserving their green coloration. The cleaned sprouts were then dried in a mechanical hot-air dryer at 40–45 °C for 2–3 days, until they reached approximately 70–80% dryness, with visible retention of their green color. The dried sprouts were pulverized using a laboratory grinder and stored under controlled humidity (60%) and temperature (20 °C) conditions until further use.

For extraction, 10 g of the dried powder was suspended in 100 mL of distilled water and incubated at 37 °C for 1 h to allow for the enzymatic conversion of glucoraphanin to sulforaphane. The suspension was then stirred at 25 °C for 3 h and filtered using Whatman No. 1 filter paper. The resulting aqueous extract was lyophilized and reconstituted in a solvent mixture of high-performance liquid chromatography (HPLC)-grade methanol and water (1:1, *v*/*v*) to a final concentration of 10 mg/mL. This solution was filtered through a 0.22 μm PTFE membrane filter (Agilent Technologies, Santa Clara, CA, USA) prior to LC-MS/MS analysis.

Analysis was performed using a Vanquish UHPLC system coupled with an Orbitrap Exploris 120 mass spectrometer (Thermo Fisher Scientific, Waltham, MA, USA) [29]. Chromatographic separation was conducted using a Waters Acquity UPLC HSS T3 column (4.6 × 100 mm, 1.8 μm, Waters, Milford, MA, USA) maintained at 40 °C, with a flow rate of 0.2 mL/min and an injection volume of 4 μL. The gradient mobile phase consisted of solvent A (0.1% formic acid in water) and solvent B (0.1% formic acid in acetonitrile) buffering with 0.01% ammonia applied as follows: 8–15% B (0–4 min), 15–32% B (4–8 min), 32–53% B (8–11 min), 53–100% B (11–24 min), held for 3 min, then returned to 8% B over 1 min for re-equilibration. The MS conditions were set as follows: spay voltage for positive (3500 V) and negative (2500 V); sheath gas (N_2_) 50; aux gas (N_2_) 15; ion transfer tube at 320 °C; vaporizer at 350 °C; HCD collision energies (%) at 15, 30, and 60; Orbitrap resolution at 120,000; scan range 100–1500 *m*/*z*. Orbitrap calibration was performed by infusing the calibration solution and optimizing spray conditions until a spray stability of ≤15% RSD was achieved before initiating the calibration routine, in which the calibration peaks were detected using positive ion mode, including caffeine, hexamethoxyphosphazene, MRFA, hexakis(2,2-difluoroethoxy)phosphazene, and hexakis(2,2,3,3-tetrafluoropropoxy)phosphazene. The UHPLC-MS/MS conditions were based on our previously validated method [41].

#### 4.3.2. LC-MS/MS and Compound Annotation

Raw Orbitrap MS/MS data files were processed using MZmine version 3.9.0 [42] prior to uploading to the GNPS platform (University of California, San Diego, La Jolla, CA, USA) [43]. Further analysis was conducted using MS-DIAL software (Riken Center for Sustainable Resource Science, Yokohama City, Kanagawa, Japan). Peaks were detected in chromatograms based on characteristic precursor ions and their fragmentation patterns. The threshold was set as follows: MS1: 0.0005 Da; MS2: 0.0001; score cut-off: 700 (0.7). Compound identification was achieved through the FBMN, which utilizes isotope patterns and mass duplication to match features against multiple online mass spectral databases, including GNPS and other American repositories [44]. FBMN was visualized by using the Cytoscape (Version 3.10.1, Institute for Systems Biology (ISB), Seattle, WA, USA) program. In the FBMN, nodes represent molecule attributions, and edges display similar relationships based on shared features. A similarity matrix was generated, and the cosine score factor was applied to retain structurally relevant connections and visualization. Compound pairs were considered significantly similar and were used to construct the edges of the molecular network [43].

### 4.4. In Vitro Cell Proliferation Assay

Cell proliferation assays were performed using HaCaT keratinocytes, CCD-986sk dermal fibroblasts, and HDP cells (PromoCell GmbH, Heidelberg, Baden-Württemberg, Germany) to compare the biological activities of minoxidil, SFN, and BSE. Cells were seeded at a density of 2.5 × 10^3^ cells per well in 100 µL of appropriate growth medium using 96-well flat-bottom tissue culture plates (SPL Life Sciences Co., Ltd., Pocheon-si, Gyeonggi-do, Republic of Korea; Product Code: P00000VA). HaCaT and CCD-986sk cells were cultured in Dulbecco’s Modified Eagle’s Medium (DMEM; Welgene Inc., Gyeongsan-si, Gyeongsangbuk-do, Republic of Korea) supplemented with 10% (*v*/*v*) fetal bovine serum (FBS; HyClone^TM^ Characterized FBS, U.S. Origin; Cytiva, Logan, UT, USA; Cat. No. SH30919.03) and 1% (*v*/*v*) penicillin–streptomycin solution (Cytiva, Logan, UT, USA; 10,000 U/mL penicillin and 10,000 µg/mL streptomycin), resulting in final concentrations of 100 U/mL penicillin and 100 µg/mL streptomycin. HDP cells were maintained in Follicle Dermal Papilla Cell Growth Medium (HFDPC) (PromoCell GmbH, Heidelberg, Baden-Württemberg, Germany), which was prepared by supplementing HFDPC Basal Medium with the manufacturer’s Supplement Pack (Cat. No. C-39620). This Supplement Pack, after mixing, comprised 4% (*v*/*v*) fetal calf serum (FCS; PromoCell GmbH; Cat. No. C-37350), 4% (*v*/*v*) bovine pituitary extract (BPE; PromoCell GmbH; Cat. No. C-39021), 5 µg/mL recombinant human insulin (PromoCell GmbH; Cat. No. C-30100; expressed in *Saccharomyces cerevisiae*), and 1 ng/mL recombinant human fibroblast growth factor-basic (rhFGF-b; PromoCell GmbH; Cat. No. C-30310; expressed in *Escherichia coli*) as the final concentration. Both recombinant proteins were of cell culture-grade with >95% purity and endotoxin levels < 1.0 EU/µg, as specified by the manufacturer.

Cells were incubated for 24 h at 37 °C in a humidified atmosphere containing 5% (*v*/*v*) CO_2_ and then serum-starved for an additional 24 h under the same conditions. Following serum starvation, cells were treated with minoxidil, SFN, or BSE at concentrations ranging from 0.01 to 100 µg/mL. After 24 h of treatment, 10 µL of Cell Proliferation Reagent WST-1 (Roche Diagnostics GmbH, Mannheim, Baden-Württemberg, Germany; Cat. No. 11644807001), a ready-to-use tetrazolium salt solution dissolved in phosphate-buffered saline (PBS), was added to each well containing 100 µL of culture medium supplemented with 0.5% (*v*/*v*) FBS or FCS, yielding a final dilution of 1:10, in accordance with the manufacturer’s recommended working concentration. After incubation for 2 h at 37 °C, absorbance was measured at 450 nm using a Multiskan^TM^ FC Microplate Photometer (Model 1510; Thermo Fisher Scientific Inc., Waltham, MA, USA). Cell viability was expressed as a percentage relative to untreated control cells [45,46].

### 4.5. In Vitro Scratch Wound Recovery Assay

To evaluate the effects of BSE on HDP cell proliferation and migration, an in vitro scratch wound recovery assay was conducted using the Incucyte^®^ S3 Live-Cell Analysis System (Model: S3 HD/2CLR System PAC; Sartorius AG, Göttingen, Niedersachsen, Germany). HDP cells were seeded at a density of 2.5 × 10^4^ cells per well in 100 µL of HDP growth medium—comprising 4% (*v*/*v*) FCS, 4% (*v*/*v*) BPE, 1 ng/mL rhFGF-b, and 5 µg/mL recombinant human insulin (all from PromoCell GmbH, Heidelberg, Baden-Württemberg, Germany)—onto 96-well Incucyte^®^ ImageLock plates (Cat. No. BA-045855; Sartorius AG, Göttingen, Niedersachsen, Germany). After incubation for 72 h at 37 °C in a humidified 5% CO_2_ atmosphere to allow monolayer formation, uniform scratch wounds approximately 700–800 µm wide were created using the Incucyte^®^ WoundMaker Tool (Model 4563; Sartorius AG), following the manufacturer’s instructions. Detached cells were removed by washing twice with PBS (pH 7.4). Cells were then treated with 100 µL of HDP medium containing 0.5% (*v*/*v*) FCS and supplemented with minoxidil, SFN, or BSE at concentrations of 0.1, 1, or 25 µg/mL. All treatments were performed in triplicate, including a vehicle control. The plates were returned to the Incucyte^®^ system, and wound images were automatically captured every 4 h over a 24 h period. Wound closure was quantified using the Incucyte^®^ Scratch Wound Analysis Software Module, which calculates relative wound density (%) by comparing cell density within the wound area to that of the surrounding monolayer, providing an objective measure of cell migration and proliferation over time.

To evaluate the migratory ability of HDP cells, a Boyden chamber assay was performed. HDP cells were serum-starved for 24 h in a medium containing 0.5% (*v*/*v*) FCS. Subsequently, cells were seeded at a density of 6 × 10^4^ cells/well into the upper chambers of polyethylene terephthalate (PET) cell culture inserts with an 8 μm pore size (Corning, New York, NY, USA), in HDP medium containing minoxidil, SFN, or BSE at concentrations of 0.1, 1, or 25 µg/mL (BSE concentrations were based on SFN equivalence). The lower chambers were filled with 600 µL of HDP medium supplemented with 4% FCS, 4% bovine pituitary extract, 1 ng/L recombinant human fibroblast growth factor, and 5 µg/L recombinant human insulin. After a 24 h incubation period to allow for migration, cells were fixed with 4% paraformaldehyde (BL539A, Biosharp, Hefei, Anhui, China) for 15 min and stained using Crystal Violet Staining Solution (C0121, Beyotime, Shanghai, China) for 10 min. Non-migrated cells on the upper side of the insert membrane were carefully removed using a cotton swab [47]. To quantify migrated cells, the basal chambers were filled with 500 µL of calcein-AM-based cell dissociation solution. Cells were incubated for 1 h at 37 °C, and the fluorescence of the dissociated calcein-AM was measured using a microplate reader at an excitation wavelength of 485 nm and an emission wavelength of 520 nm [48].

### 4.6. In Vivo Oral Absorption in Rats

To compare the plasma concentration profiles of commercially available pure SFN and SFN-rich BSE, a pharmacokinetic study was conducted in rats. Free SFN (5 mg/kg, solubilized in 0.05% (*w*/*v*) sodium carboxymethyl cellulose [NaCMC]) or BSE, corresponding to SFN doses of 5, 10, and 20 mg/kg (dissolved in water to a final volume of 800 µL), was administered orally. To evaluate absolute oral bioavailability, an intravenous (IV) bolus dose of SFN (2 mg/kg, dissolved in normal saline, 200 µL) was administered via a cannulated femoral vein. Blood samples (300 µL) were collected from the femoral artery at predetermined time points: 0.17, 0.5, 1, 1.5, 2, 4, 8, and 12 h post-IV dosing, and 0.17, 0.5, 1, 2, 4, 6, 8, and 12 h post-oral dosing. Samples were immediately centrifuged at 13,000× *g* for 5 min at 4 °C to separate plasma, which was stored at −80 °C until further analysis.

The plasma concentrations of SFN were determined using liquid chromatography–tandem mass spectrometry (LC–MS/MS) following protein precipitation and solid-phase extraction. Briefly, 180 µL of plasma was spiked with 10 µL of SFN working solution (0.2–20 µg/mL) and 10 µL of internal standard (IS), phenethyl isothiocyanate (PHE, 5 µg/mL in acetonitrile). Proteins were precipitated by adding 200 µL of a solvent mixture consisting of 25% acetonitrile, 25% water, and 50% ethanol, followed by centrifugation at 18,340× *g* for 5 min at 4 °C. The resulting 400 µL supernatant was subjected to SPE using Oasis^®^ HLB 96-well µElution plates (2 mg sorbent per well, 30 µm particle size; Waters Corporation, Milford, MA, USA), preconditioned with 200 µL each of methanol and water. After sample loading, the wells were washed sequentially with 200 µL of water and 50% aqueous methanol, and analytes were eluted with 400 µL of acetonitrile (2 × 200 µL).

Chromatographic separation was achieved on an ACQUITY UPLC system (Waters Corporation) using a BEH C_18_ column (50 × 2.1 mm, 1.7 µm). The mobile phase consisted of 10 mM ammonium acetate in water (30%, solvent A) and 0.1% formic acid in acetonitrile (70%, solvent B), delivered at a flow rate of 0.6 mL/min. The column temperature was maintained at 50 °C, and the injection volume was 10 µL.

Detection was performed using a Xevo TQ-S triple quadrupole mass spectrometer (Waters Corporation) equipped with an electrospray ionization source in the positive-ion mode. Multiple reaction monitoring (MRM) transitions were set at *m*/*z* 178.0 → 114.0 for SFN (cone voltage: 24 V; collision energy: 30 eV) and *m*/*z* 164.0 → 130.0 for PHE (cone voltage: 23 V; collision energy: 22 eV). The instrument settings included a capillary voltage of 3.0 kV, source temperature of 120 °C, desolvation temperature of 500 °C, desolvation gas (nitrogen) flow of 800 L/h, and cone gas (nitrogen) flow of 50 L/h. Argon was used as the collision gas. All the MRM parameters were optimized using the software IntelliStart^TM^ (Waters Corporation).

The calibration curve for SFN was linear over the range of 0.5–1000 ng/mL, with a lower limit of detection (LOD) of 0.5 ng/mL. The overall recovery of SFN using the described extraction method was 90 ± 3%, when compared to the direct analysis of the standard diluted in methanol.

### 4.7. In Vivo Hair Regrowth Efficacy

To evaluate the hair regrowth efficacy of BSE, an AGA model was established in male C57BL/6J mice via the subcutaneous injection of testosterone (0.5 mg/kg/day) in the dermal area for five consecutive days [49,50]. The untreated control group did not receive testosterone. On day 6, dorsal hair was removed from all mice. All mice were anesthetized using 3% isoflurane in 100% oxygen at a flow rate of 0.8 L/min, and the suppression of deep pedal reflexes was confirmed in the mice. Then, the desired dorsal region was shaved using electric clippers, followed by the application of rosin wax. After 2 min, the rosin wax was removed with the help of adhesive tape. The application of rosin wax was repeated one more time to completely remove the few remaining hairs [51].

Testosterone-treated mice were then randomly assigned to six groups (*n* = 6 per group). The first group served as the negative control and only received the vehicle. The second group was treated topically once daily with 100 µL of 1% minoxidil (vehicle composed of ethanol, propylene glycol, and water [60:20:20, *v*/*v*/*v*]). The third group received oral finasteride at a dose of 1 mg/kg once daily. The remaining three groups were administered oral BSE at doses equivalent to 5, 10, or 20 mg/kg (based on its own content), respectively, once daily for 15 consecutive days.

Hair regrowth was visually monitored and photographed on days 0, 3, 6, 9, 12, and 15. Regrowth progression was quantitatively assessed using the software ImageJ (version 1.54p; National Institutes of Health, Bethesda, MD, USA) by measuring the areas corresponding to telogen-phase skin (pink), anagen transition zones (black), and fully covered hair regions. On day 15, the regrown dorsal hair was trimmed and weighed using an analytical balance to assess total hair mass. To evaluate hair follicle length, mice were first anesthetized using 3% isoflurane in 100% oxygen at a flow rate of 0.8 L/min to minimize movement and distress. The dorsal skin area was then cleaned with 70% ethanol to prevent contamination. Using sterile fine-tipped forceps, approximately 5–20 hairs were grasped close to the skin surface and swiftly plucked in the direction of hair growth to minimize follicular damage. Only hair shafts with intact follicular bulbs were selected for analysis. This procedure was repeated at three distinct dorsal sites per animal to obtain a representative sampling of hair regrowth. Finally, the lengths of selected hair follicles were measured microscopically [52,53].

Blood samples were collected in heparinized tubes and centrifuged at 13,000× *g* for 5 min at 4 °C to isolate plasma. The plasma was stored at −80 °C until analysis for testosterone and DHT concentrations.

Dorsal skin tissues were harvested and divided into two sections. One portion was fixed in neutral-buffered 10% formalin (HT501640, Sigma-Aldrich, St. Louis, MO, USA; pH ~ 7.0), embedded in paraffin, and sectioned longitudinally and transversely at a thickness of 5 μm. Sections were stained with H&E following the protocol of Heo et al. (2011): 6 min in Harris hematoxylin, 1 min in running tap water, 10 min in eosin Y, followed by sequential dehydration in 70%, 95%, and 100% ethanol (1 min each), and two rinses in xylene (1 min each). Histological evaluation was conducted to assess follicle morphology and regrowth stage [54]. The other portion was immediately snap-frozen in liquid nitrogen for subsequent analysis of hair growth-related protein expression.

### 4.8. Plasma Testosterone and DHT Levels

Plasma samples collected after the final dose of once-daily treatments were used to quantify serum testosterone and DHT levels using commercial ELISA kits: the Mouse Testosterone ELISA Kit and Mouse DHT ELISA Kit (LifeSpan BioSciences, Inc., Seattle, WA, USA). All assays were conducted following the manufacturer’s protocols.

### 4.9. Western Blot Assay

To assess protein expression levels in skin and liver tissues, samples were lysed using M-PER^TM^ Mammalian Protein Extraction Reagent (Thermo Fisher Scientific) supplemented with a 1× working concentration of Xpert Protease Inhibitor Cocktail Solution (GenDEPOT, Barker, TX, USA), which included 50 μM PMSF, 10 μM Pepstatin A, 20 μM Leupeptin, 100 μM Benzamidine, and 50 μM Bestatin. The lysates were incubated on ice for 30 min and centrifuged at 12,000× *g* for 10 min at 4 °C to remove debris. The supernatants were collected, and total protein concentrations were determined using the Pierce^TM^ BCA Protein Assay Kit (Thermo Fisher Scientific).

Equal amounts of protein (20–30 μg) were loaded onto 10% SDS–polyacrylamide gels and electrophoresed using a Bio-Rad Mini-PROTEAN Tetra System with the Tris–Glycine–SDS running buffer. A pre-stained molecular weight marker (Precision Plus Protein^TM^ Dual Color Standards, Bio-Rad) was used as a reference. Proteins were then transferred to PVDF membranes (Immobilon^®^-P, Millipore, Burlington, MA, USA) using the wet transfer method with the Tris–Glycine buffer containing 20% methanol at 0.2 A for 120 min at 4 °C.

Membranes were blocked with 5% skim milk in TBST buffer (20 mM Tris–HCl, 150 mM NaCl, 0.2% Tween 20, pH 7.4) for 1 h at room temperature and incubated overnight at 4 °C with one of the following primary antibodies diluted at 1:1000: anti-HSP90 (Santa Cruz Biotechnology, sc-13119, Dallas, TX, USA), anti-β-catenin (Cell Signaling Technology, #8480S, Danvers, MA, USA), anti-Lef-1 (Cell Signaling Technology, #2230S, Danvers, MA, USA), anti-Akr1c21 (Invitrogen, PA5-36572, Carlsbad, CA, USA), or anti-Dhrs9 (MyBioSource, MBS9125625, San Diego, CA, USA). Each antibody was applied to separate membranes to avoid cross-reactivity and ensure target specificity.

After primary antibody incubation, membranes were washed four times for 10 min each with TBST at 50 rpm on a shaker, followed by incubation with HRP-conjugated secondary antibodies (Thermo Fisher Scientific) diluted 1:3000 for 1 h at room temperature. The membranes were then washed three times for 5 min each with TBST under the same conditions. Protein bands were visualized using the enhanced chemiluminescence (ECL) reagent (Bio-Rad) and imaged using the iBright^TM^ CL1500 Imaging System (Thermo Fisher Scientific). Densitometric analysis was performed using the software ImageJ (NIH), and band intensities were normalized to HSP90 expression to ensure consistent protein quantification across all samples.

### 4.10. Molecular Docking

The 3D structures of the β-catenin (PDB ID: 1JDH) and Akr1c2 (PDB ID: 1IHI) proteins were obtained from the RCSB Protein Data Bank (https://www.rcsb.org; accessed on 21 April 2025). Protein and ligand structures were prepared using MGL Tools 1.5.6 (The Scripps Research Institute, La Jolla, CA, USA) [55]. The receptor structures were processed by removing water molecules, adding polar hydrogen atoms, and assigning Kollman charges. The structures of major compounds (1, 2, 6, 9, 16, 18, 24, 57, 60, finasteride, minoxidil, and iso-ursodeoxycholic acid) were downloaded from PubChem (https://pubchem.ncbi.nlm.nih.gov; accessed on 23 April 2025) in the SDF format, whereas compounds 17 and D-sulforaphane were prepared using the Avogadro package [56] employing the MMFF94 force field method. These compound geometries were converted to the pdbqt format using Open Babel [55]. Co-crystallized ligands and selected compounds were assigned Gasteiger charges, and torsions were adjusted before applying the AutoGrid 4.0 function. The grid box coordinates were determined using PyMOL (Schrödinger, Inc., New York, NY, USA) [57] to define the centroid of the native ligand in the crystal structures or the key amino acids of the protein binding pocket. Protein–ligand docking calculations were performed using AutoDock 4.0 [58] via MGLTools 1.5.6, which employs a semi-empirical, free energy-based scoring function specifically designed for molecular docking. Molecular docking was performed by identifying the active sites of each protein. Then, the positive controls and selected compounds were processed to generate their docking functions. The grid box dimension was set to parameters including size (x, y, z): 16 × 20 × 38 Å, centered at (−1.581, 32.034, 36.549) for the Akr1c2 protein and 74 × 40 × 80 Å, centered at (7.864, 10.930, 29.134) for the β-catenin protein. The scoring function was based on van der Waals interactions, electrostatics, hydrogen bonding, desolvation effects, and torsional entropy contributions, enabling the accurate prediction of ligand-binding modes and affinities. The protocol’s accuracy was validated by applying the same method for the native ligands. Briefly, the root mean square deviation values between the original and redocked native ligands were 0.1013 Å (PDB ID: 1IHI), indicating the reliability and accuracy of the method. Residue–ligand interactions were visualized with Discovery Studio 2021 (BIOVIA, San Diego, CA, USA).

### 4.11. Molecular Dynamic Simulation

To examine whether the docked complex remains stable over time in a simulated biological environment (e.g., aqueous solvent, body temperature, ionic conditions), molecular dynamics (MD) simulations were performed using GROMACS 2023.3 (KTH Royal Institute of Technology, Stockholm, Sweden) [59]. The AmberGS [60] force field was selected during topology preparation, along with the TIP3P water model [61]. Hydrogen and charges were added to both protein and ligand structures. The system was solvated using the SPC/E water model [62] in a cubic simulation box, with a 1 nm buffer distance between the protein and the box boundary. Energy minimization was conducted using the steepest descent algorithm for 50,000 steps. The system was then equilibrated under NVT (number of particles, volume, and temperature) conditions at 300 K using a V-rescale thermostat with a time constant of 0.1 ps to stabilize the temperature [63]. NPT (constant number of particles, pressure, and temperature) equilibration was performed using the Berendsen barostat to adjust the system pressure to 1 bar. After removing positional restraints on the solute, production MD simulations were carried out for 20,000 picoseconds (ps) under NPT conditions using the Parrinello–Rahman barostat. Trajectory analyses included the calculations of the complex backbone RMSD, RMSF, SASA, H-bonds, and Rg values.

### 4.12. Statistical Analysis

All data are presented as mean ± standard deviation (SD) or standard error of mean (SEM), as specified. Statistical significance was defined as *p* < 0.05. Differences among three or more groups were analyzed using one-way analysis of variance followed by Tukey’s post hoc test.

## 5. Conclusions

This study highlights the potential of BSE, enriched with SFN, as a natural therapeutic candidate for AGA. BSE significantly enhanced cell proliferation and migration in hair follicle-related cells, demonstrating superior in vitro efficacy compared to minoxidil and pure SFN. In an AGA mouse model, the oral administration of BSE induced dose-dependent hair regrowth, achieving up to 99% recovery at a dose of 20 mg/kg, comparable to that of finasteride. Hair follicle length, density, and weight also increased significantly following BSE treatment. Mechanistically, BSE enhanced androgen metabolism by upregulating DHT-degrading enzymes Akr1c21 and Dhrs9, and it activated the Wnt/β-catenin signaling pathway through the increased expression of β-catenin and Lef-1 in both liver and skin tissues. Pharmacokinetic analysis confirmed the sustained plasma exposure of SFN after BSE administration. Moreover, the repeated oral dosing of BSE showed no signs of hepatotoxicity or intestinal damage, supporting its safety profile. In silico studies further corroborated these effects by demonstrating favorable binding affinities and the dynamic interactions of major BSE components identified by the GNPS-FBMN analysis. Collectively, our findings suggest that BSE promotes hair regrowth via the dual modulation of follicular signaling and androgen metabolism, offering a promising and safe alternative to conventional AGA treatments.

## Figures and Tables

**Table 1:** Pharmacokinetic parameters of SFN in rats after intravenous (IV) or oral administration of SFN or BSE.

Test Material	SFN-IV	SFN-Oral	BSE (5)	BSE (10)	BSE (20)
Administration route	IV	Oral	Oral	Oral	Oral
SFN dose (mg/kg)	2	5	5	10	20
T_max_ (h)		2 ± 0	4 ± 0	4 ± 0	4 ± 0
T_1/2_ (h)	7 ± 3	5 ± 0	7 ± 1	14 ± 8	13 ± 2
C_max_ (ng/mL)	339 ± 50	155 ± 17	101 ± 3	206 ± 13	344 ± 18
AUC_last_ (ng·h/mL)	403 ± 52	874 ± 46	794 ± 73	1475 ± 17	2082 ± 27
AUC_inf_ (ng·h/mL)	421 ± 67	934 ± 41	897 ± 71	1997 ± 403	2650 ± 125
Bioavailability (%)	100	87 ± 5	79 ± 7	73 ± 1	52 ± 1

Values are presented as mean ± SD (*n* = 3). T_max_, time to reach maximum plasma concentration of SFN; T_1/2_, plasma half-life of SFN; C_max_, maximum plasma SFN concentration; AUC_last_, area under the plasma concentration–time curve between zero and the last measurable plasma concentration; AUC_inf_, area under the plasma concentration–time curve between zero and infinity. Bioavailability (%) = (AUC_last, oral_/Dose_SFN, oral_)/(AUC_last, IV_/Dose_SFN, IV_) × 100.

## Data Availability

The original contributions presented in this study are included in the article and Supplementary Materials and indicated as representative data. The raw data supporting the conclusions of this article will be made available by the authors upon request, which should be directed to the corresponding authors.

## Supplementary Materials

The following supporting information can be downloaded at https://www.mdpi.com/article/10.3390/ijms26157467/s1.

## Abbreviations

The following abbreviations are used in this manuscript:

AGA	Androgenetic alopecia
FDA	Food drug administration
Akr1c2	Aldo-keto reductase family 1 member C2
AUC_inf_	Area under the curve to infinity
AUC_last_	Area under the curve at the last time point
BSE	Broccoli sprout extract
CCD-986sk	Dermal fibroblast
C_max_	Maximum plasma concentration
Dhrs9	Dehydrogenase/reductase 9
DHT	Dihydrotestosterone
FBMN	Feature-based molecular networking
GNPS	Global natural products social molecular networking
HaCaT	Keratinocytes
HDP	Human dermal papilla
HSD	Hepatic hydroxysteroid dehydrogenase
IV	Intravenous
Lef-1	Lymphoid enhancer-binding factor-1
Rg	Radius of gyration
RMSD	Root mean square deviation
SASA	Solvent-accessible surface area
SFN	Sulforaphane
SMSF	Root mean square fluctuation
T_1/2_	Half-life
T_max_	Time to maximum peak drug concentration
