# Supplementary material for: Sulforaphane-Rich Broccoli Sprout Extract Promotes Hair Regrowth in an Androgenetic Alopecia Mouse Model via Enhanced Dihydrotestosterone Metabolism
Source: Int J Mol Sci. 2025 Aug 1;26(15):7467. doi: 10.3390/ijms26157467 (PMC12347391; doi:10.3390/ijms26157467)
Supplement: Supplementary file 1 [file ijms-26-07467-s001.zip › ijms-3717073-supplementary.pdf]

Supplementary Materials

Sulforaphane-Rich Broccoli Sprout Extract Promotes Hair Regrowth in an Androgenetic Alopecia Mouse Model via Enhanced Dihydrotestosterone Metabolism

Laxman Subedi [1,2,†], Duc Dat Le [3,4,†], Eunbin Kim [1], Susmita Phuyal [1], Arjun Dhwoj Bamjan [1], Vinhquang Truong [3,4], Nam Ah Kim [1,5], Jung-Hyun Shim [1,5], Jong Bae Seo [1], Suk-Jung Oh [6], Mina Lee [3,4,*] and Jin Woo Park [1,5,*]

1 Department of Biomedicine, Health & Life Convergence Sciences, BK21 Four, Biomedical and Healthcare Research Institute, Mokpo National University, Jeonnam 58554, Republic of Korea; laxmansubedi789@gmail.com (L.S.); dmsqls0749@naver.com (E.K.); sushmitaphuyal54@gmail.com (S.P.); arjun.bamjan@gmail.com (A.D.B.); namahk87@mnu.ac.kr (N.A.K.); s1004jh@gmail.com (J.-H.S.); jbseo@mnu.ac.kr (J.B.S.); jwpark@mokpo.ac.kr (J.W.P.)

2 Biomedicine Cutting Edge Formulation Technology Center, Mokpo National University, Jeonnam 58554, Republic of Korea; laxmansubedi789@gmail.com (L.S.)

3 College of Pharmacy and Research Institute of Life and Pharmaceutical Sciences, Sunchon National University, Jeonnam 57922, Republic of Korea; ddle@scnu.ac.kr (D.D.L.); quangvtruong00@gmail.com (V.T.); minalee@scnu.ac.kr (M.L.)

4 Department of Natural Cosmetics Science and Smart Beautytech Research Institute, Sunchon National University, Jeonnam 57922, Republic of Korea; ddle@scnu.ac.kr (D.D.L.); quangvtruong00@gmail.com (V.T.); minalee@scnu.ac.kr (M.L.)

5 College of Pharmacy and Natural Medicine Research Institute, Mokpo National University, Jeonnam 58554, Republic of Korea; namahk87@mnu.ac.kr (N.A.K.); s1004jh@gmail.com (J.-H.S.); jwpark@mokpo.ac.kr (J.W.P.)

6 Research & Development, Ecoworld Pharm Co. Ltd., Jeonnam 57304, Republic of Korea; sj.oh@ecoworldpharm.com (S.-J.O.)

† These authors contributed equally to this work.

* Correspondence: minalee@scnu.ac.kr (M.L.); jwpark@mokpo.ac.kr (J.W.P.)

Table S1. Identification of components from BSE sample.

No.	Compound	*m/z* (Da)	RT (min)	Formula	Adduct	Error (ppm)	Confidence level (max: 1000)	Class
1	Arginine	175.1186	1.216	$C_6H_{14}N_4O_2$	$[M+H]^+$	-2.01	848	Amino acids
2	L-proline	116.0703	1.307	$C_5H_9NO_2$	$[M+H]^+$	-2.63	986	Amino acids
3	Glutamic acid	148.0602	1.267	$C_5H_9NO_4$	$[M+H]^+$	-1.58	944	Amino acids
4	Unknown	309.1283	1.323	-	$[M+H]^+$	-	-	Unknowns
5	Unknown	381.0784	1.290	-	$[M+H]^+$	-	-	Unknowns
6	Valine	118.0859	1.490	$C_5H_{11}NO_2$	$[M+H]^+$	-3.01	895	Amino acids
7	Cytidine	244.0925	1.490	$C_9H_{13}N_3O_5$	$[M+H]^+$	-1.22	885	Alkaloids
8	Unknown	487.1772	1.499	-	$[M+H]^+$	-	-	Unknowns
9	Adenine	136.0615	1.589	$C_5H_5N_5$	$[M+H]^+$	-2.00	1000	Alkaloids
10	Pregabalin	160.1329	1.509	$C_8H_{17}NO_2$	$[M+H]^+$	-1.91	955	Alkaloids
11	Guanine	152.0562	1.652	$C_5H_5N_5O$	$[M+H]^+$	-3.20	905	Alkaloids
12	Unknown	262.1275	1.652	-	$[M+H]^+$	-	-	Unknowns
13	Uracil	113.0343	1.904	$C_4H_4N_2O_2$	$[M+H]^+$	2.25	952	Alkaloids
14	Unknown	260.1602	2.004	-	[M+H]+	-	-	Unknowns
15	Unknown	260.1959	2.004	-	[M+H]+	-	-	Unknowns

Table S1. Cont.

No.	Compound	*m/z* (Da)	RT (min)	Formula	Adduct	Error (ppm)	Confidence level (max: 1000)	Class
16	Adenosine	268.1030	2.004	$C_{10}H_{13}N_5O_4$	$[M+H]^+$	-3.84	988	Alkaloids
17	[6,10a-dihydroxy-4-(hydroxymethyl)-4,7,11b-trimethyl-9-oxo-1,2,3,4a,5,6,6a,7,11,11a-decahydronaphtho[2,1-f][1]benzofuran-5-yl] acetate	431.2033	2.054	$C_{22}H_{32}O_7$	$[M+Na]^+$	1.68	892	Organic acids
18	Norleucine	132.1014	2.195	$C_6H_{13}NO_2$	$[M+H]^+$	-3.82	1000	Amino acids
19	Tyrosine	182.0808	2.054	$C_9H_{11}NO_3$	$[M+H]^+$	-2.03	879	Amino acids
20	Stearic acid amide	284.2942	2.206	$C_{10}H_{13}N_5O_5$	$[M+H]^+$	-2.08	848	Fatty acids
21	Unknown	293.1488	3.155	-	$[M+NH_4]^+$	-	-	Unknowns
22	Unknown	359.2406	3.146	-	$[M+H]^+$	-	-	Unknowns
23	Indoline	120.0805	3.808	C_8H_9N	$[M+H]^+$	-2.30	922	Alkaloids
24	Phenylalanine	166.0859	3.808	$C_9H_{11}NO_2$	$[M+H]^+$	-2.14	853	Amino acids

Table S1. Cont.

No.	Compound	*m/z* (Da)	RT (min)	Formula	Adduct	Error (ppm)	Confidence level (max: 1000)	Class
25	Serylleucine	219.1333	4.264	$C_9H_{18}N_2O_4$	$[M+H]^+$	-2.89	775	Amino acids
26	Pterosin G	235.1324	4.287	$C_{14}H_{18}O_3$	$[M+H]^+$	-2.00	883	Sesquiterpenoids
27	Unknown	299.0544	4.298	-	$[M+H]^+$	-	-	Unknowns
28	Alanyl-Leucine	203.1386	4.571	$C_9H_{18}N_2O_3$	$[M+H]^+$	-2.06	787	Amino acids
29	Threonylleucine	233.1491	4.581	$C_{10}H_{20}N_2O_4$	$[M+H]^+$	-2.07	810	Amino acids
30	Aspartylphenylalanine	281.1129	4.633	$C_{13}H_{16}N_2O_5$	$[M+H]^+$	-1.06	758	Amino acids
31	Leucyl-valine	231.1698	4.757	$C_{11}H_{22}N_2O_3$	$[M+H]^+$	-2.25	893	Amino acids
32	(*Z*)-9,12,13-trihydroxyoctadec-15-enoic acid	353.2293	4.716	$C_{18}H_{34}O_5$	$[M+Na]^+$	-1.54	979	Fatty acids
33	γ-Glutamylleucine	261.1441	4.879	$C_{11}H_{20}N_2O_5$	$[M+H]^+$	-1.52	813	Amino acids
34	Isoleucylglutamate	261.1439	4.879	$C_{11}H_{20}N_2O_5$	$[M+H]^+$	-2.29	861	Amino acids
35	Unknown	286.1753	4.922	-	$[M+Na]^+$	-	-	Unknowns
36	Unknown	319.1355	4.869	-	$[M+H]^+$	-	-	Unknowns
37	Leucylproline	229.1539	5.440	$C_{11}H_{20}N_2O_3$	$[M+H]^+$	-3.36	926	Amino acids
38	Unknown	253.1177	5.429	-	$[M+H]^+$	-	-	Unknowns

Table S1. Cont.

No.	Compound	*m/z* (Da)	RT (min)	Formula	Adduct	Error (ppm)	Confidence level (max: 1000)	Class
39	4-Amino-2-[[2-(methoxymethyl)pyrrolidin-1-yl]methyl]oxolan-3-ol	231.1698	5.813	$C_{11}H_{22}N_2O_3$	$[M+H]^+$	-2.25	912	Alkaloids
40	Salidroside	323.1093	5.832	$C_{14}H_{20}O_7$	$[M+Na]^+$	-2.55	1000	Glycosides
41	Unknown	354.0666	5.844	-	$[M+H]^+$	-	-	Unknowns
42	Tryptophan	205.0969	5.866	$C_{11}H_{12}N_2O_2$	$[M+H]^+$	-1.24	975	Amino acids
43	D-pantothenic acid	220.1175	6.114	$C_9H_{17}NO_5$	$[M+H]^+$	-2.04	850	Organic acids
44	Unknown	316.2223	6.135	-	$[M+H]^+$	-	-	Unknowns
45	2-[(2-amino-3-methylbutanoyl)amino]-3-phenylpropanoic acid	265.1539	7.004	$C_{14}H_{20}N_2O_3$	$[M+H]^+$	-2.90	998	Alkaloids
46	Catechin	291.0858	6.447	$C_{15}H_{14}O_6$	$[M+H]^+$	-1.77	893	Flavonoid
47	Syringin	395.1305	6.475	$C_{17}H_{24}O_9$	$[M+Na]^+$	-1.91	935	Glycosides
48	Unknown	318.1555	6.993	$C_{14}H_{20}O_7$	$[M+H]^+$	-	-	Unknowns
49	Leucyl-Leucine	245.1856	7.083	$C_{12}H_{24}N_2O_3$	$[M+H]^+$	-1.51	822	Amino acids
50	Sinapic acid	225.0753	7.509	$C_{11}H_{12}O_5$	$[M+H]^+$	-2.00	989	Organic acids
51	Unknown	310.1643	7.538	-	$[M+H]^+$	-	-	Unknowns
52	Unknown	164.0193	8.117	-	$[M+H]^+$	-	-	Unknowns

Table S1. Cont.

No.	Compound	*m/z* (Da)	RT (min)	Formula	Adduct	Error (mDa)	Confidence level (max: 1000)	Class
53	Indole-3-carboxylic acid	162.0547	8.458	$C_9H_7NO_2$	$[M+H]^+$	-1.57	891	Organic acids
54	Leucyl-phenylalanine	279.1699	8.752	$C_{15}H_{22}N_2O_3$	$[M+H]^+$	-1.50	950	Amino acids
55	Unknown	678.2744	8.709	-	$[M+H]^+$	-	-	Unknowns
56	Unknown	576.2865	8.947	-	$[M+H]^+$	-	-	Unknowns
57	Silodosin	496.2430	9.158	$C_{25}H_{32}F_3N_3O_4$	$[M+H]^+$	2.48	966	Alkaloids
58	Unknown	374.1443	9.581	-	$[M+H]^+$	-	-	Unknowns
59	Unknown	561.2724	9.558	-	$[M+H]^+$	-	-	Unknowns
60	L-Sulforaphane	178.0351	10.164	$C_6H_{11}NOS_2$	$[M+H]^+$	-2.15	880	Alkaloids
61	(2*R*)-6-(2-hydroxyethyl)-2,5,7-trimethyl-2,3-dihydroinden-1-one	219.1375	10.229	$C_{14}H_{18}O_2$	$[M+H]^+$	-2.08	995	Sesquiterpenoids
62	Unknown	336.1068	10.397	$C_{14}H_{19}NO_7$	$[M+Na]^+$	-	-	Unknowns
63	Unknown	498.2592	10.498	-	$[M+H]^+$			Unknowns
64	Valsartan	458.2164	10.663	$C_{24}H_{29}N_5O_3$	$[M+Na]^+$	0.30	907	Alkaloids
65	Sibiricose A6	531.1700	11.191	$C_{23}H_{32}O_{15}$	$[M+H-H_2O]^+$	-1.57	992	Glycosides
66	Unknown	772.2658	11.191	-	$[M+NH_4]^+$	-	-	Unknowns

Table S1. Cont.

No.	Compound	*m/z* (Da)	RT (min)	Formula	Adduct	Error (ppm)	Confidence level (max: 1000)	Class
67	Diethyltoluamide	192.0508	12.361	$C_{12}H_{17}NO$	$[M+H]^+$	-2.03	815	Alkaloids
68	Unknown	978.3208	12.383	-	$[M+H]^+$	-	-	Unknowns
69	Unknown	358.0887	13.184	-	$[M+H]^+$	-	-	Unknowns
70	Unknown	404.0643	14.055	-	$[M+H]^+$	-	-	Unknowns
71	Nandrolone	275.2001	15.604	$C_{18}H_{26}O_2$	$[M+H]^+$	-1.66	973	Terpenes
72	(9*S*,13*R*)-12-Oxo phytodienoic acid	293.2103	15.604	$C_{18}H_{28}O_3$	$[M+H]^+$	-2.80	976	Fatty acids
73	7-(2-methoxyethyl)-1,3-dimethyl-8-[(4-methylpiperazin-1-yl)methyl]purine-2,6-dione	351.2136	15.604	$C_{16}H_{26}N_6O_3$	$[M+H]^+$	-0.90	735	Alkaloids
74	4-Methoxy-2H-isoquinolin-1-one	176.0702	16.233	$C_{10}H_9NO_2$	$[M+H]^+$	-2.30	965	Alkaloids
75	2-(6-Methyl-6-hydroxyheptyl)furan-2(5H)-one	195.1373	16.693	$C_{12}H_{20}O_3$	$[M-H_2O+H]^+$	-3.36	916	Furan
76	Diphenylphosphate	251.0462	21.566	$C_{12}H_{11}O_4P$	$[M+H]^+$	-2.28	828	Organic phosphate
77	Estra-1,3,5(10)-triene-3,17-diol	273.1843	21.566	$C_{18}H_{24}O_2$	$[M+H]^+$	-2.22	875	Terpenes

Table S2. Comparison of spectra between identified components and public data.

No.	Compound	*m/z* (Da)	RT (min)	Formula	Adduct	Error (ppm)	Confidence level (max: 1000)	Chromatogram
1	Arginine	175.1186	1.216	$C_6H_{14}N_4O_2$	$[M+H]^+$	-2.01	848	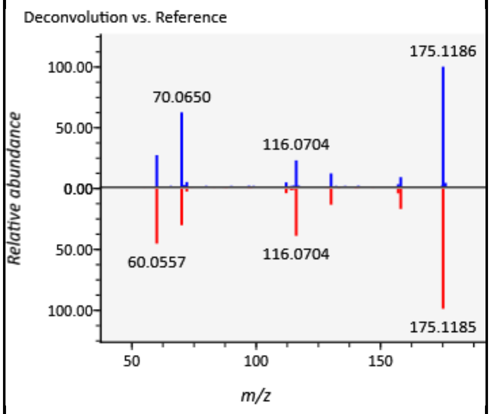
2	L-proline	116.0703	1.307	$C_5H_9NO_2$	$[M+H]^+$	-2.63	986	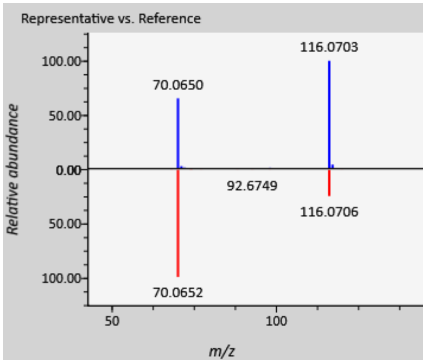
3	Glutamic acid	148.0602	1.267	C5H9NO4	$[M+H]^+$	-1.58	944	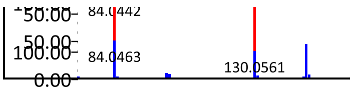
4	Unknown	309.1283	1.323	-	$[M+H]^+$	-	-	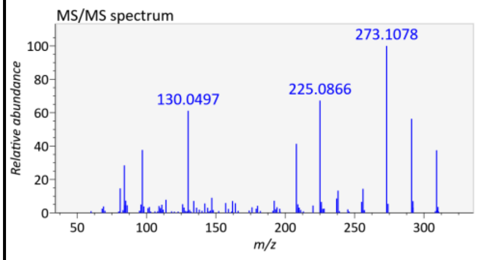
5	Unknown	381.0784	1.290	-	$[M+H]^+$	-	-	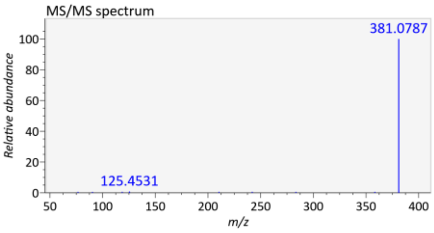

6	Valine	118.0859	1.490	$C_5H_{11}NO_2$	$[M+H]^+$	-3.01	895	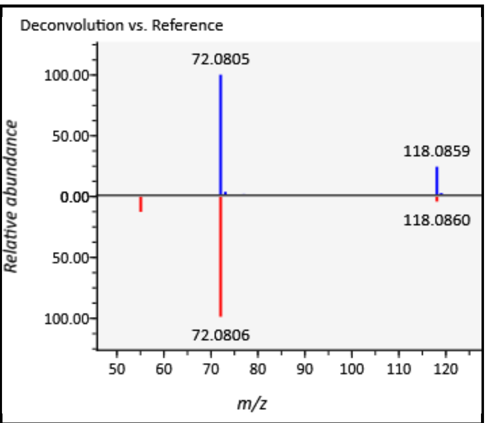
7	Cytidine	244.0925	1.490	$C_9H_{13}N_3O_5$	$[M+H]^+$	-1.22	885	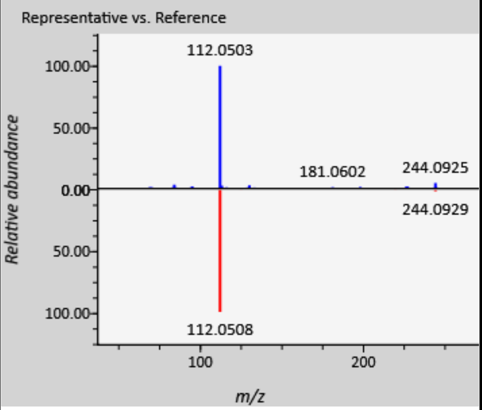
8	Unknown	487.1772	1.499	-	$[M+H]^+$	-	-	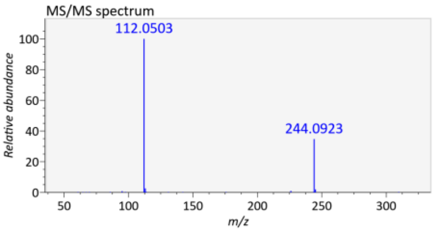
9	Adenine	136.0615	1.589	$C_5H_5N_5$	$[M+H]^+$	-2.00	1000	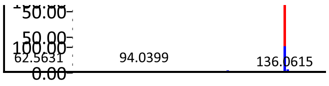
10	Pregabalin	160.1329	1.509	$C_8H_{17}NO_2$	$[M+H]^+$	-1.91	955	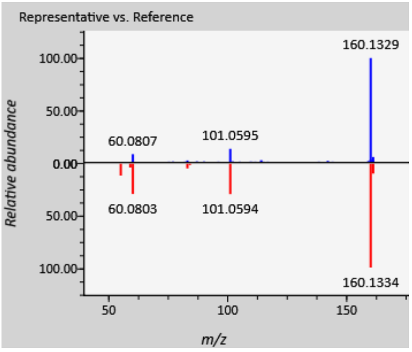

11	Guanine	152.0564	1.652	C5H5N5O	$[M+H]^+$	-3.20	905	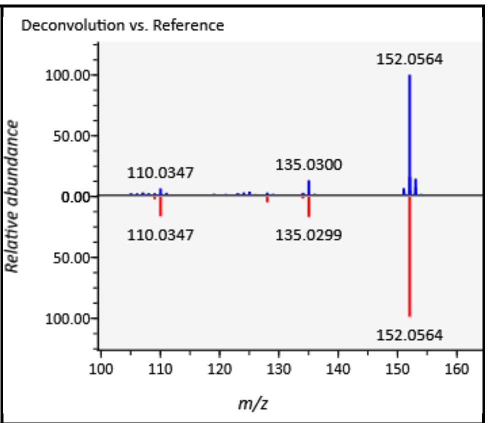
12	Unknown	262.1275	1.652	-	$[M+H]^+$	-	-	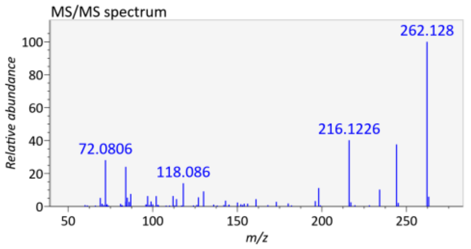
13	Uracil	113.0343	1.904	C4H4N2O2	$[M+H]^+$	2.25	952	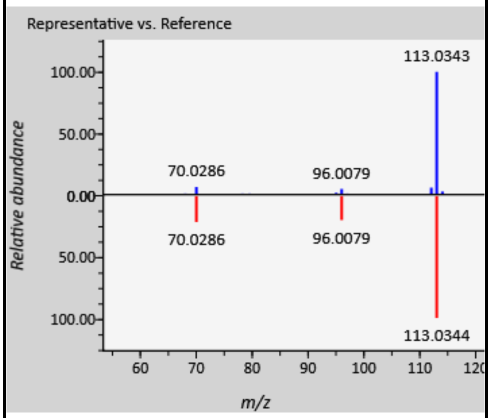
14	Unknown	260.1602	2.004	-	[M+H]+	-	-	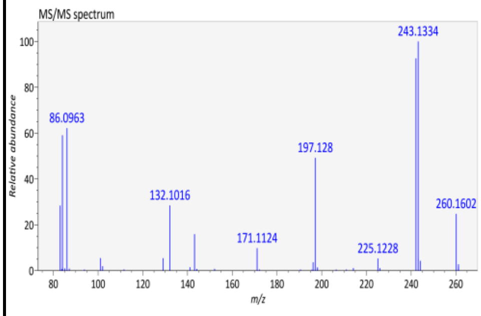
15	Unknown	260.1959	2.004	$C_{14}H_{16}N_2O_3$	[M+H]+	-	-	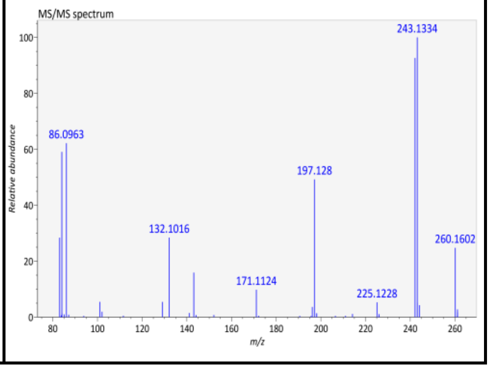

16	Adenosine	268.1030	2.004	$C_{10}H_{13}N_5O_4$	$[M+H]^+$	-3.84	988	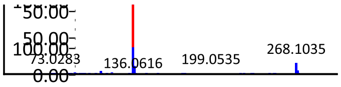
17	[6,10a-dihydroxy-4-(hydroxymethyl)-4,7,11b-trimethyl-9-oxo-1,2,3,4a,5,6,6a,7,11,11a-decahydronaphtho[2,1-f][1]benzofuran-5-yl] acetate	431.2033	2.054	C22H32O7	$[M+Na]^+$	1.68	892	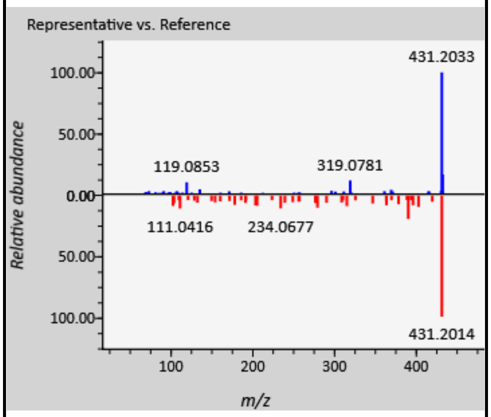
18	Norleucine	132.1016	2.195	$C_6H_{13}NO_2$	$[M+H]^+$	-3.82	1000	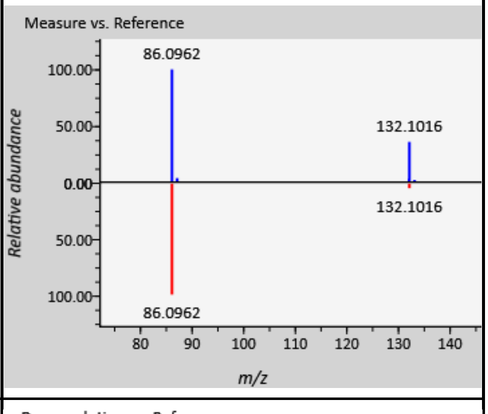
19	Tyrosine	182.0808	2.054	$C_9H_{11}NO_3$	$[M+H]^+$	-2.03	879	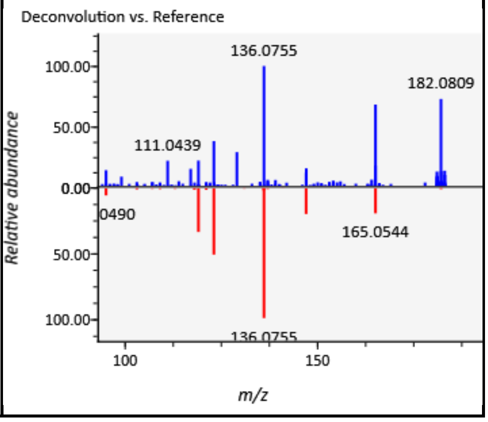

20	Stearic acid amide	284.2942	2.206	$C_{18}H_{37}NO$	$[M+H]^+$	-2.08	848	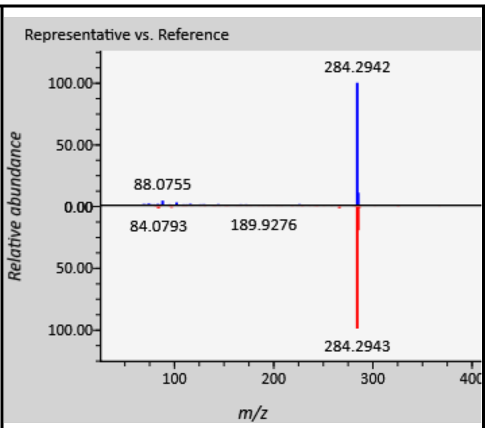
21	Unknown	293.1488	3.155	-	$[M+NH_4]^+$	-	-	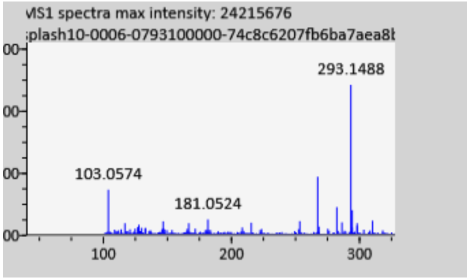
22	Unknown	359.2406	3.146	-	$[M+H]^+$	-	-	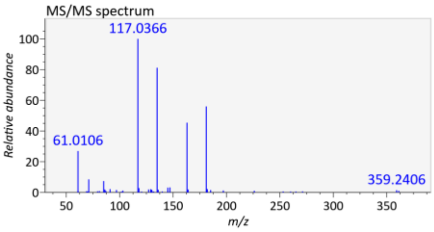
23	Indoline	120.0805	3.808	C_8H_9N	$[M+H]^+$	-2.30	922	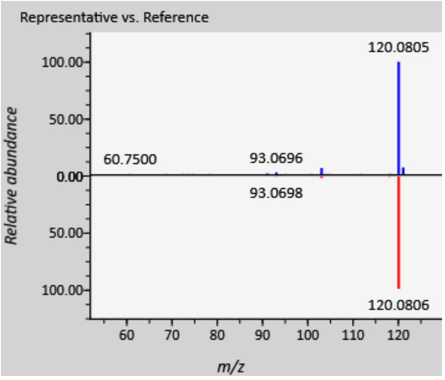
24	Phenylalanine	166.0859	3.808	$C_9H_{11}NO_2$	$[M+H]^+$	-2.14	853	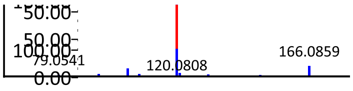

25	Serylleucine	219.1333	4.264	$C_9H_{18}N_2O_4$	$[M+H]^+$	-2.89	775	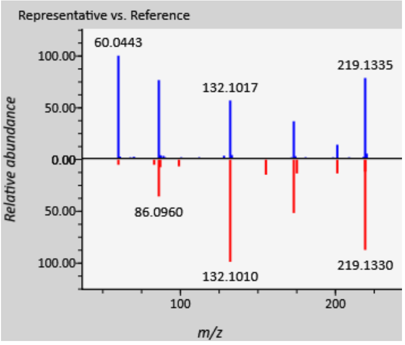
26	Pterosin G	235.1324	4.287	$C_{14}H_{18}O_3$	$[M+H]^+$	-2.00	883	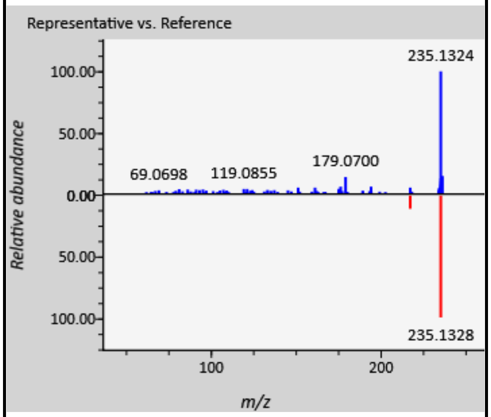
27	Unknown	299.0544	4.298	-	$[M+H]^+$	-	-	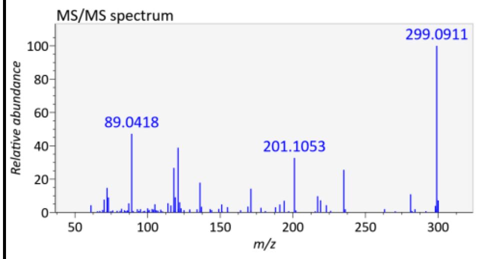
28	Alanyl-Leucine	203.1386	4.571	$C_9H_{18}N_2O_3$	$[M+H]^+$	-2.06	787	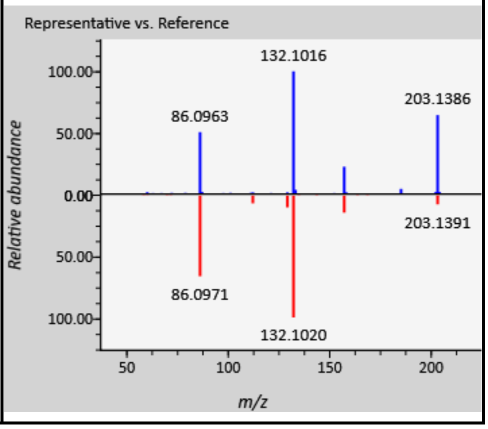

29	Threonylleucine	233.1491	4.581	$C_{10}H_{20}N_2O_4$	$[M+H]^+$	-2.07	810	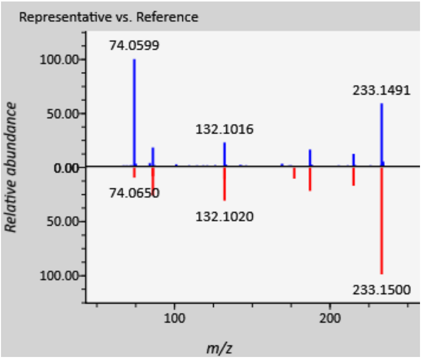
30	Aspartylphenylal anine	281.1129	4.633	$C_{13}H_{16}N_2O_5$	$[M+H]^+$	-1.06	758	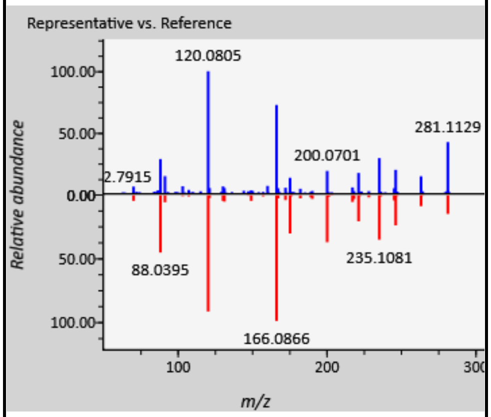
31	Leucyl-Valine	231.1698	4.757	-	$[M+H]^+$	-2.25	893	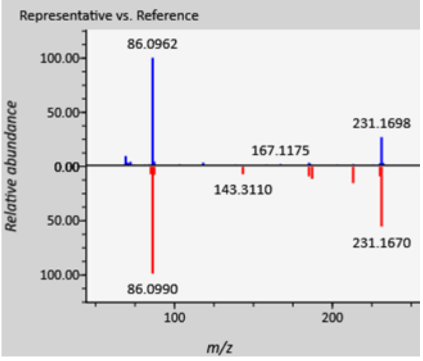
32	(Z)-9,12,13-trihydroxyoctadec-15-enoic acid	353.2293	4.716	$C_{18}H_{34}O_5$	$[M+Na]^+$	-1.54	979	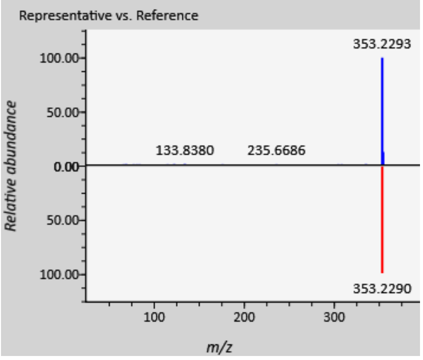

33	γ-Glutamylleucine	261.1441	4.879	C11H20N2O5	[M+H]$^+$	-1.52	813	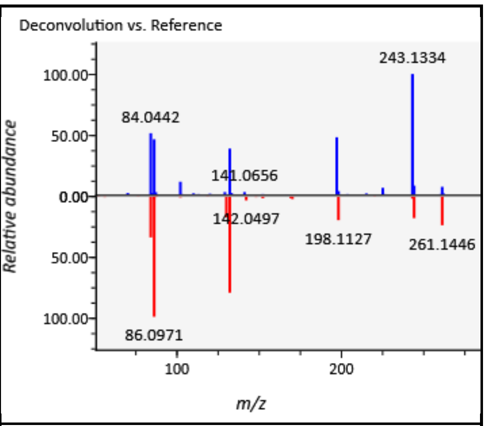
34	Isoleucylglutamate	261.1439	4.879	C11H20N2O5	[M+H]$^+$	-2.29	861	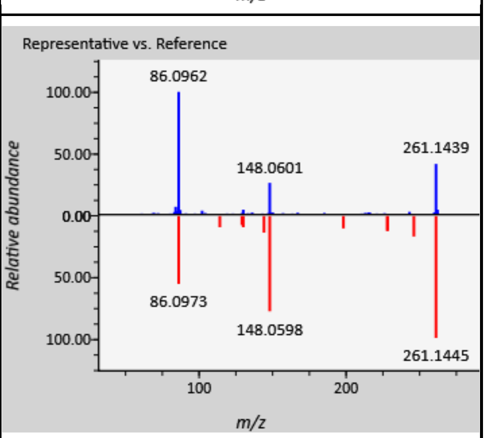
35	Unkmown	286.1753	4.911	-	[M+Na]$^+$	-	-	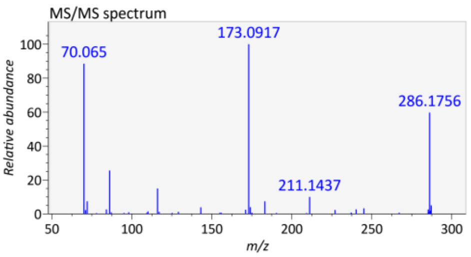
36	Unknown	319.1355	4.869	-	[M+H]$^+$	-	-	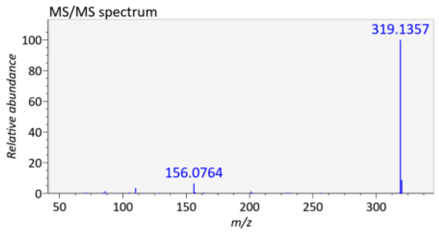
37	Leucylproline	229.1539	5.440	$C_{11}H_{20}N_2O_3$	[M+H]$^+$	-3.36	926	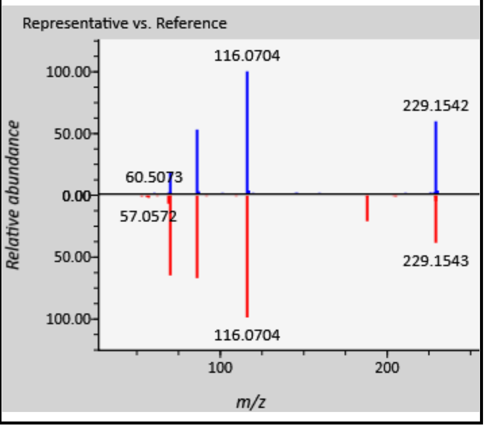

38	Unknown	253.1177	5.429	-	[M+H]$^+$	-	-	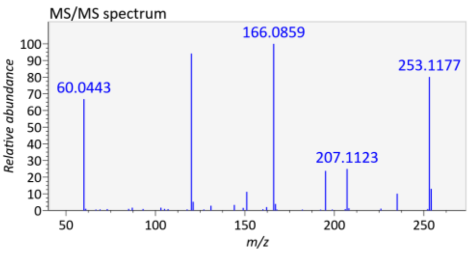
39	4-Amino-2-[[2-(methoxymethyl) pyrrolidin-1-yl]methyl]oxolan -3-ol	231.1698	5.813	$C_{11}H_{22}N_2O_3$	[M+H]$^+$	-2.25	912	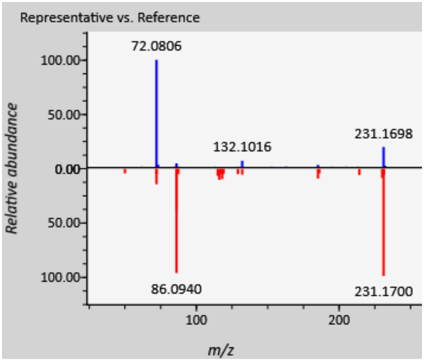
40	Salidroside	323.1093	5.832	C14H20O7	[M+Na]$^+$	-2.55	1000	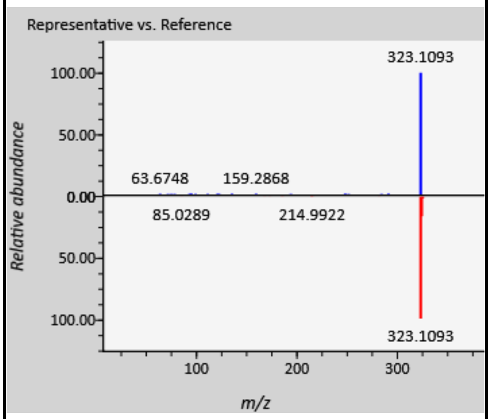
41	Unknown	354.0666	5.844	-	[M+H]$^+$	-	-	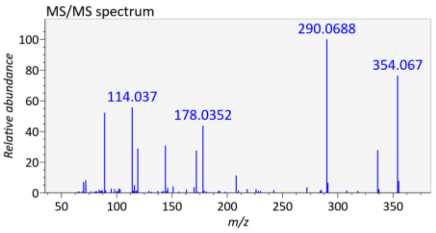
42	Tryptophan	205.0969	5.866	$C_{11}H_{12}N_2O_2$	[M+H]$^+$	-1.24	975	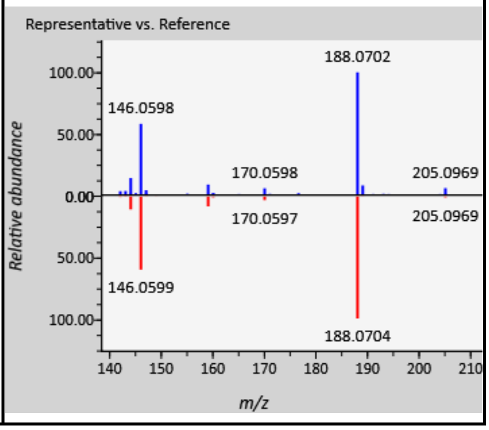

43	D-pantothenic acid	220.1175	6.114	$C_9H_{17}NO_5$	$[M+H]^+$	-2.04	850	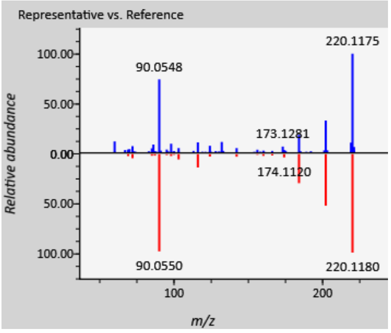
44	Unknown	316.2223	6.135	-	$[M+H]^+$	-	-	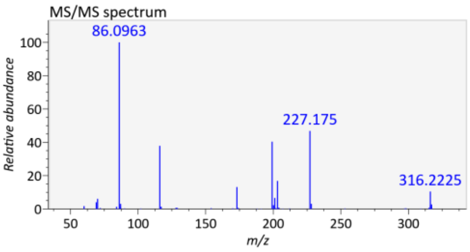
45	2-[(2-amino-3-methylbutanoyl) amino]-3-phenylpropanoic acid	265.1539	7.004	$C_{14}H_{20}N_2O_3$	$[M+H]^+$	-2.90	998	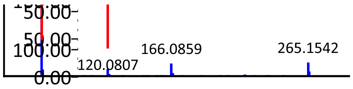
46	Catechin	291.0858	6.447	C15H14O6	$[M+H]^+$	-1.77	893	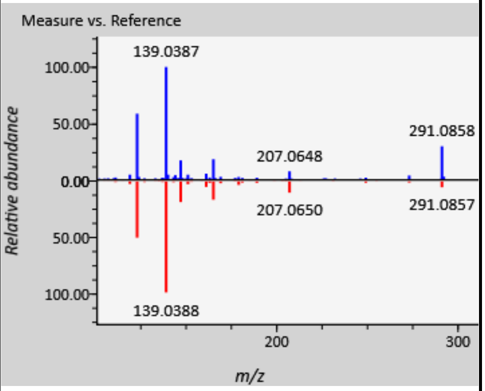
47	Syringin	395.1305	6.475	$C_{17}H_{24}O_9$	$[M+Na]^+$	-1.91	935	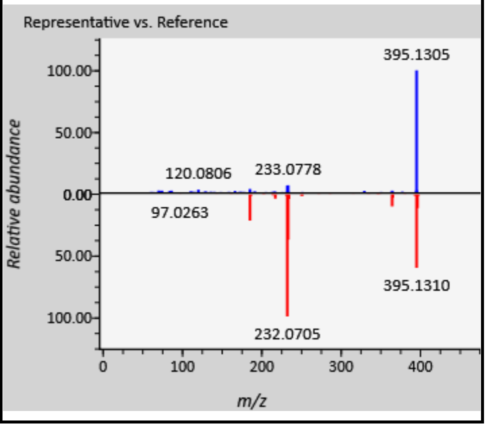

48	Unknown	318.1555	6.993	$C_{14}H_{20}O_7$	$[M+H]^+$	-	-	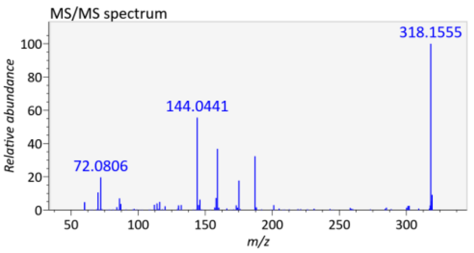
49	Leucyl-Leucine	245.1856	7.083	$C_{12}H_{24}N_2O_3$	$[M+H]^+$	-1.51	822	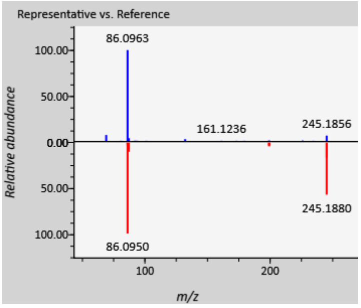
50	Sinapic acid	225.0753	7.509	C11H12O5	0.89	-2.00	989	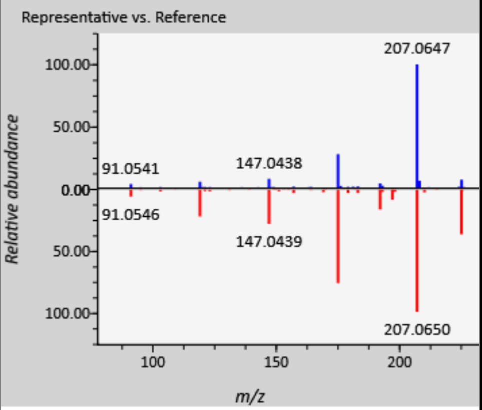
51	Unknown	310.1643	7.538	-	$[M+H]^+$	-	-	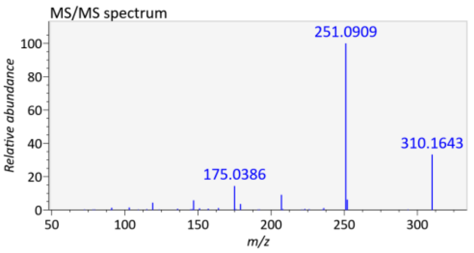
52	Unknown	164.0738	8.117	-	$[M+H]^+$	-	-	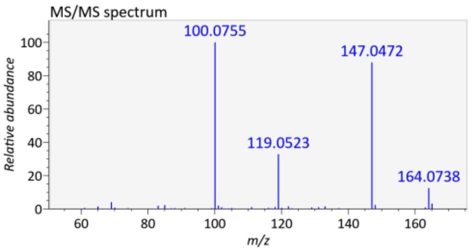

53	Indole-3-carboxylic acid	162.0547	8.458	$C_9H_7NO_2$	$[M+H]^+$	-1.57	891	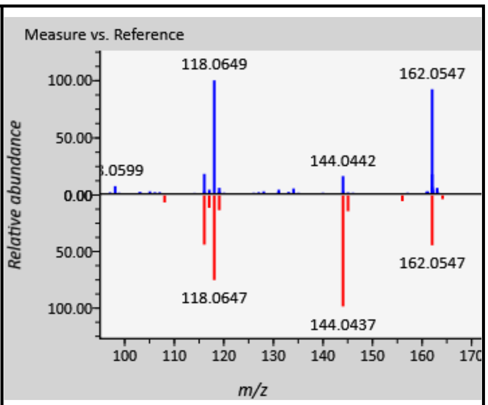
54	Leucyl-phenylalanine	279.1699	8.752	$C_{15}H_{22}N_2O_3$	$[M+H]^+$	-1.50	950	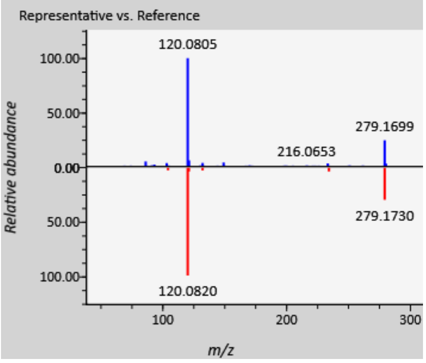
55	Unknown	678.2744	8.709	-	$[M+H]^+$	-	-	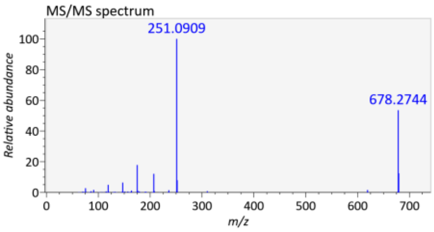
56	Unknown	576.2865	8.947	-	$[M+H]^+$	-	-	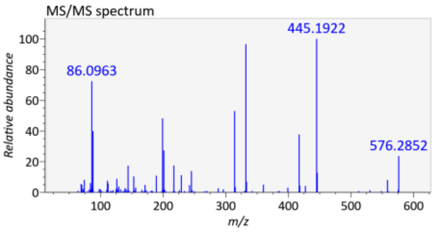
57	Silodosin	496.2430	9.158	$C_{25}H_{32}F_3N_3O_4$	$[M+H]^+$	2.48	966	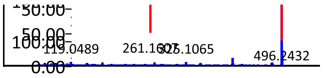

58	Unknown	374.1443	9.581	-	$[M+H]^+$	-	-	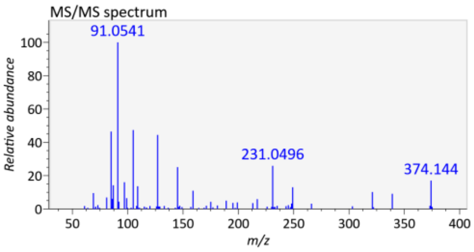
59	Unknown	561.2724	9.558	-	$[M+H]^+$	-	-	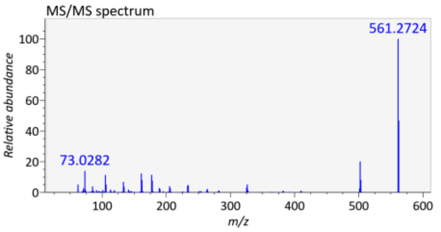
60	L-Sulforaphane	178.0351	10.164	$C_6H_{11}NOS_2$	$[M+H]^+$	-2.15	880	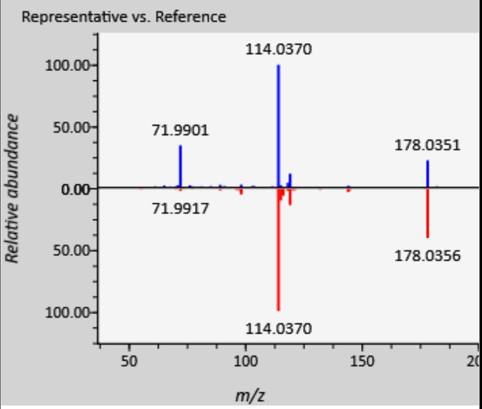
61	(2*R*)-6-(2-hydroxyethyl)-2,5,7-trimethyl-2,3-dihydroinden-1-one	219.1375	10.229	$C_{14}H_{18}O_2$	$[M+H]^+$	-2.08	995	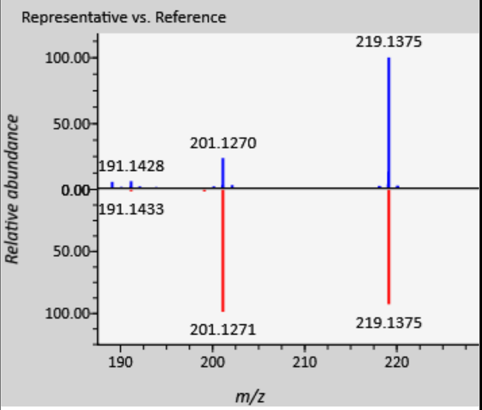
62	Unknown	336.1068	10.397	-	$[M+Na]^+$	-	-	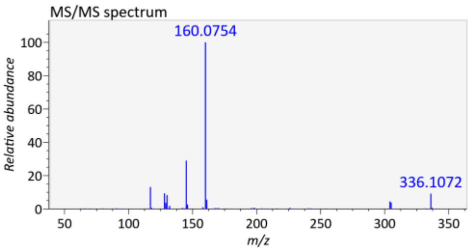

63	Unknown	498.2592	10.498	-	$[M+H]^+$	-	-	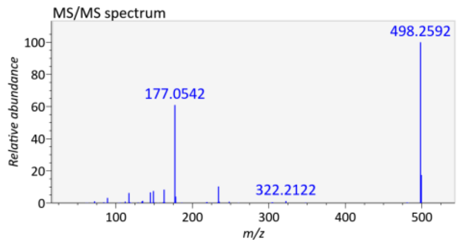
64	Valsartan	458.2164	10.663	$C_{24}H_{29}N_5O_3$	$[M+Na]^+$	0.30	907	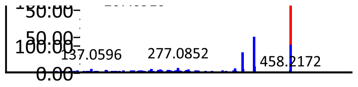
65	Sibiricose A6	531.1700	11.191	$C_{23}H_{32}O_{15}$	$[M+H-H_2O]^+$	-1.57	992	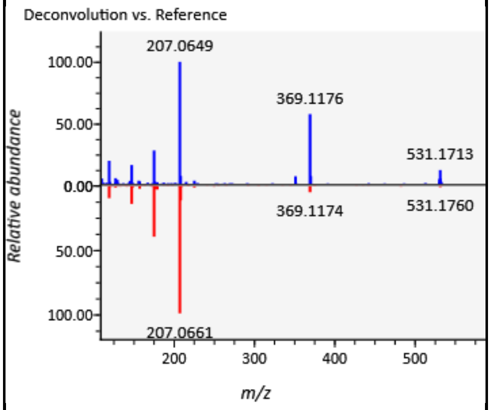
66	Unknown	772.2658	11.191	-	$[M+NH_4]^+$	-	-	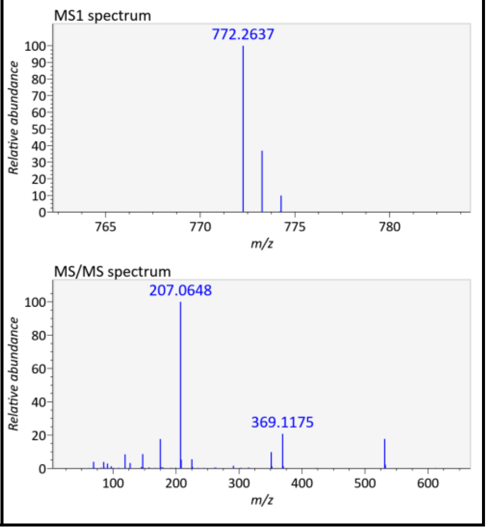

67	Diethyltoluamide	192.1379	12.361	$C_{12}H_{17}NO$	$[M+H]^+$	-2.03	815	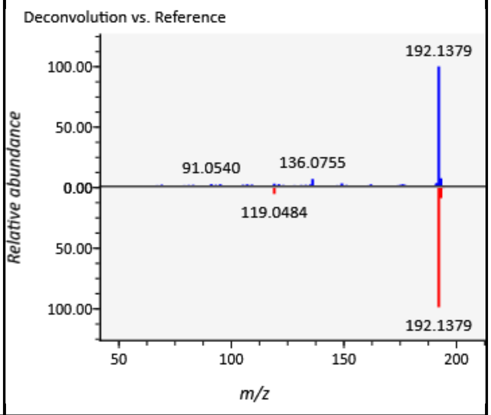
68	Unknown	978.3208	12.383	-	$[M+H]^+$	-	-	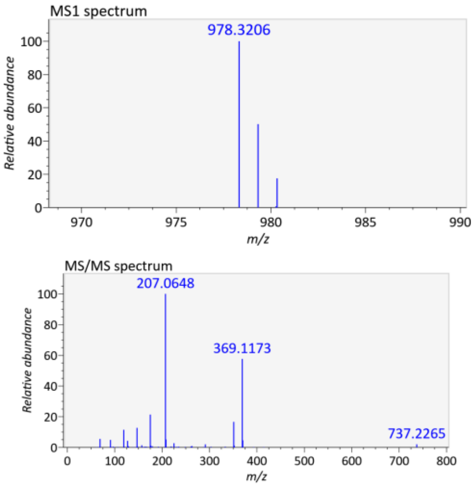
69	Unknown	358.0887	13.184	-	$[M+H]^+$	-	-	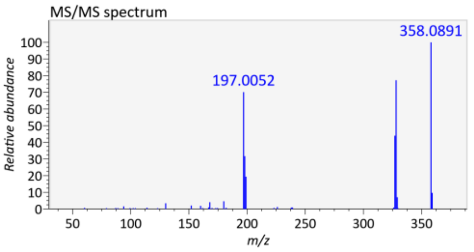
70	Unknown	404.0643	14.055	-	$[M+H]^+$	-	-	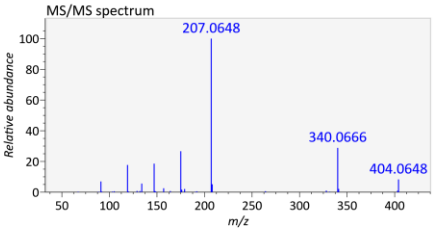

71	Nandrolone	275.2001	15.604	$C_{18}H_{26}O_2$	$[M+H]^+$	-1.66	973	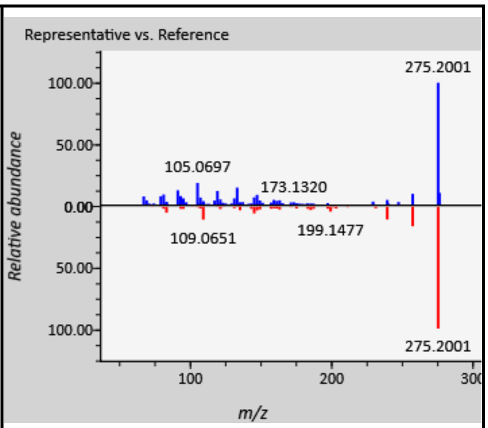
72	(9*S*,13*R*)-12-Oxo phytodienoic acid	293.2105	15.604	$C_{18}H_{28}O_3$	$[M+H]^+$	-2.80	976	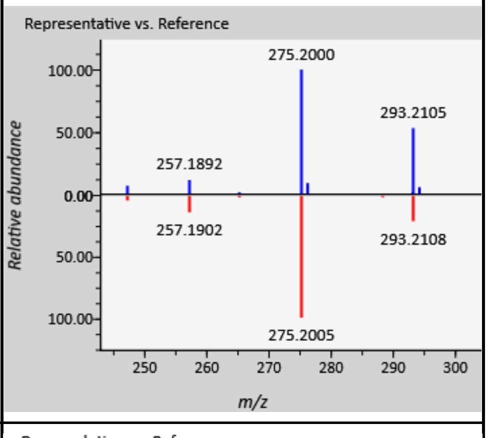
73	7-(2-methoxyethyl)-1,3-dimethyl-8-[(4-methylpiperazin-1-yl)methyl]purine-2,6-dione	351.2136	15.604	$C_{16}H_{26}N_6O_3$	$[M+H]^+$	-0.90	735	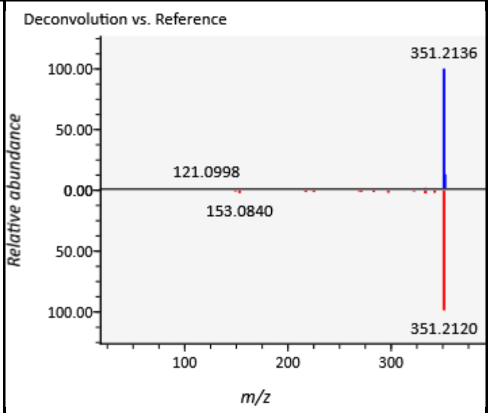
74	4-Methoxy-2h-isoquinolin-1-one	176.0702	16.233	$C_{10}H_9NO_2$	$[M+H]^+$	-2.30	965	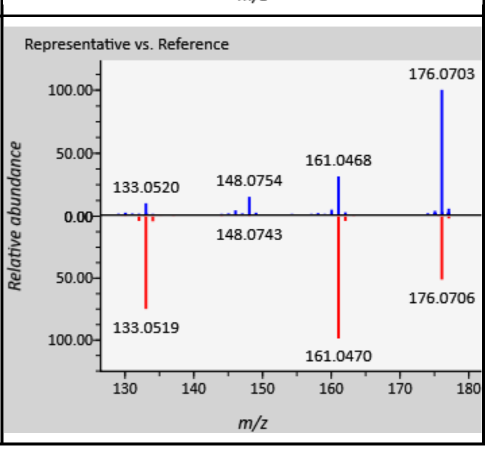

75	2-(6-Methyl-6-hydroxyheptyl)furan-2(5H)-one	195.1373	16.693	$C_{12}H_{20}O_3$	$[M-H_2O+H]^+$	-3.36	916	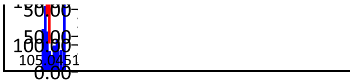
76	Diphenylphosphate	251.0462	21.566	C12H11O4P	$[M+H]^+$	-2.28	828	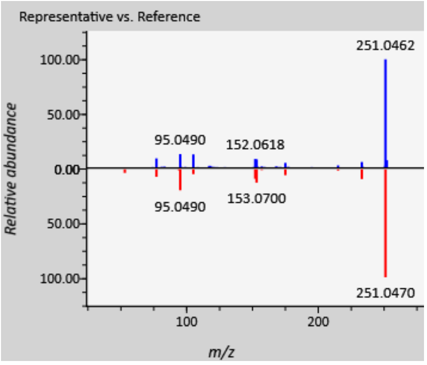
77	Estra-1,3,5(10)-triene-3,17-diol	273.1843	21.566	C18H24O2	$[M+H]^+$	-2.22	875	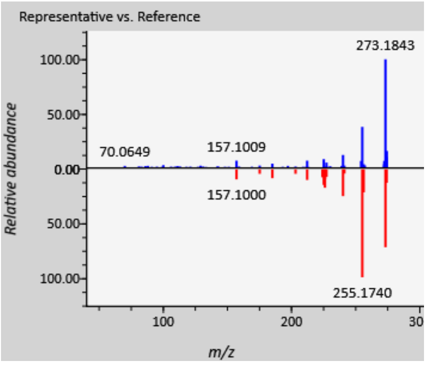

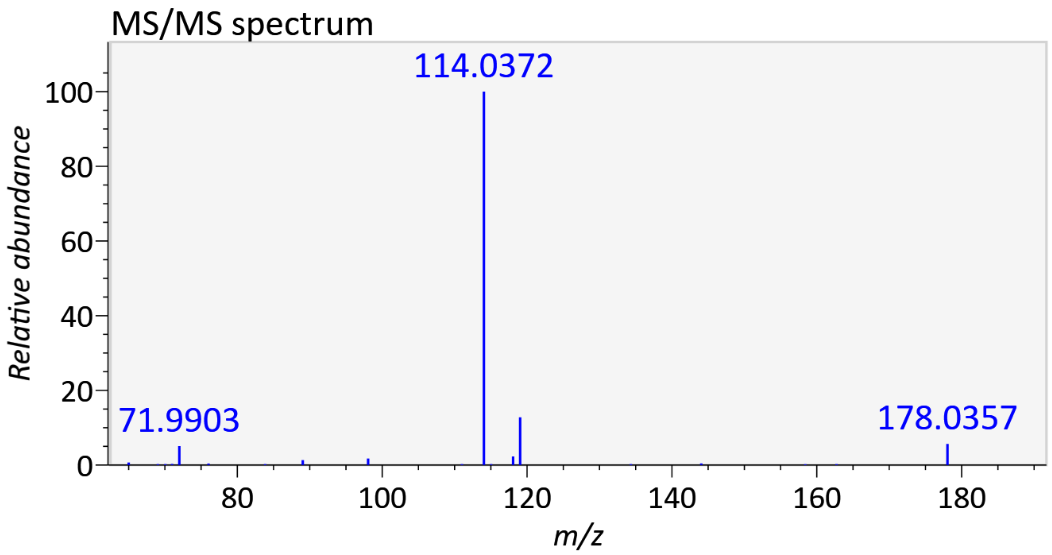

Figure S1. Mass spectrum of L-sulforaphane standard.

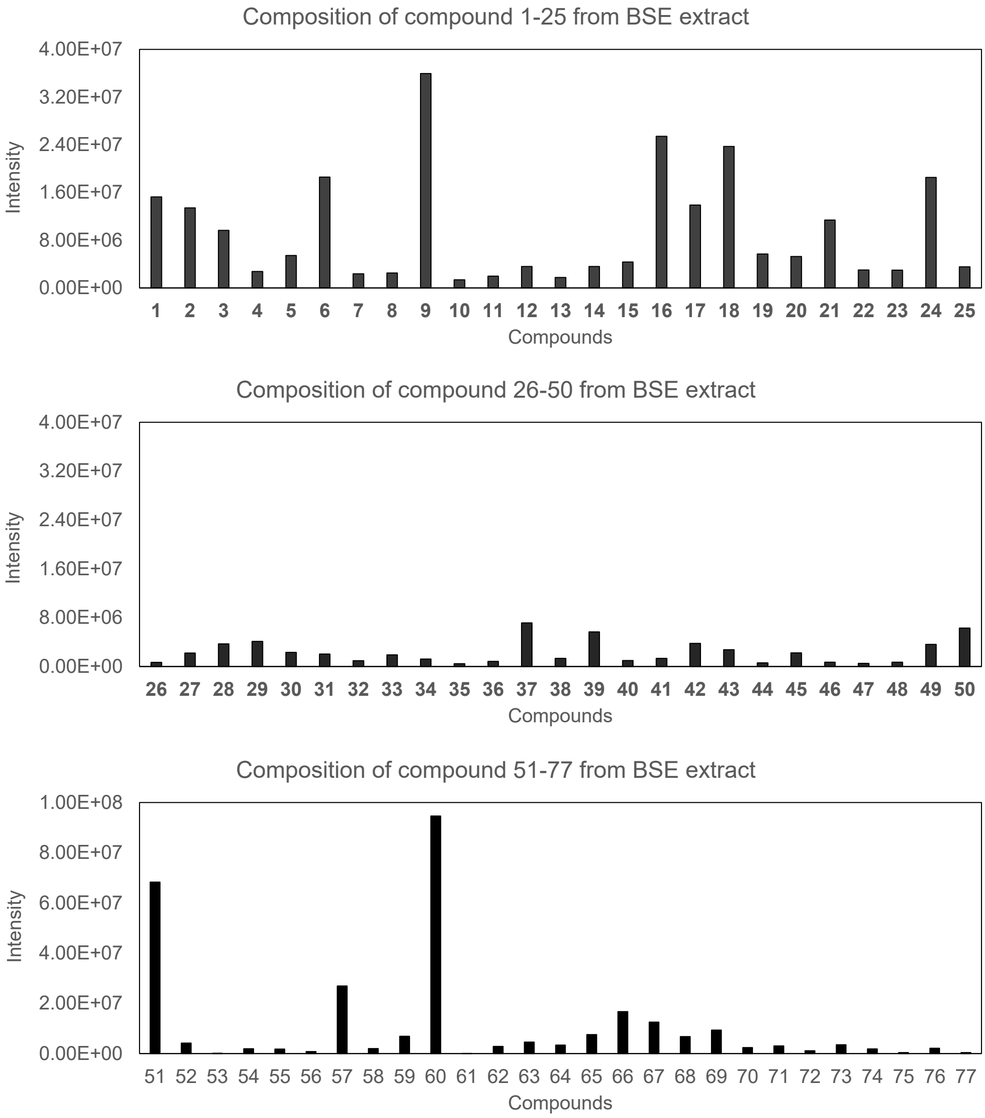

Figure S2. Chemical composition of compounds identified from BSE.

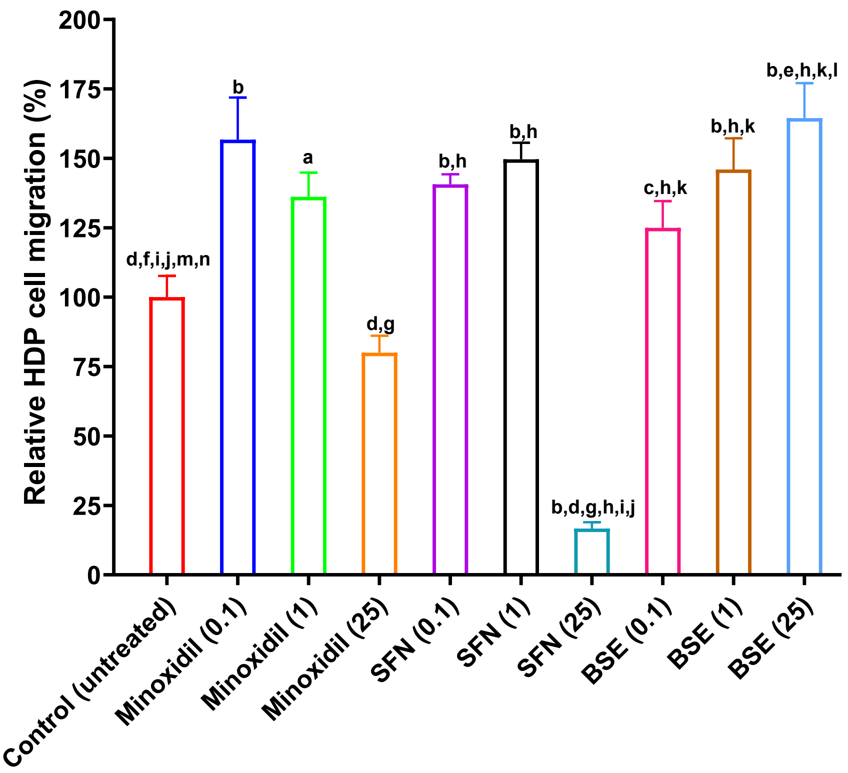

Figure S3. In vitro cell migration assay. Relative migration of HDP cells following treatment with minoxidil (0.1, 1, and 25 µg/mL), SFN (0.1, 1, and 25 µg/mL), and BSE (0.1, 1, and 25 µg/mL, based on SFN equivalence). Values are presented as mean ± SD (n = 3 per group). $^{a}p < 0.01$ and $^{b}p < 0.001$ compared to control (untreated); $^{c}p < 0.05$ and $^{d}p < 0.001$ compared to minoxidil (0.1); $^{e}p < 0.05$, $^{f}p < 0.01$, and $^{g}p < 0.001$ compared to minoxidil (1); $^{h}p < 0.001$ compared to minoxidil (25); $^{i}p < 0.001$ compared to SFN (0.1); $^{j}p < 0.001$ compared to SFN (1); $^{k}p < 0.001$ compared to SFN (25); $^{l}p < 0.01$ compared to BSE (0.1); $^{m}p < 0.001$ compared to BSE (1); $^{n}p < 0.001$ compared to BSE (25).

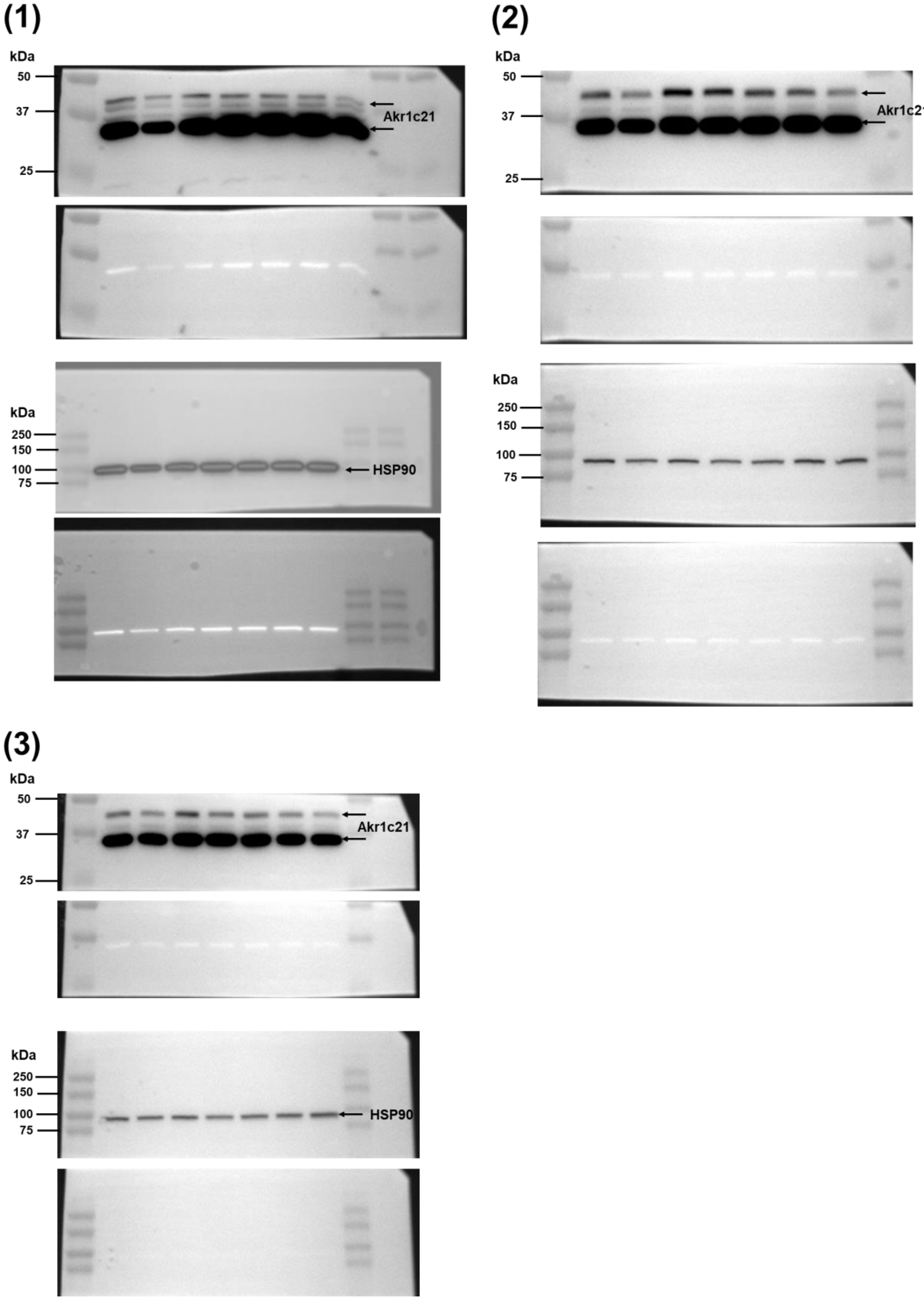

Figure S4. Western blot bands of Akr1c21 and HSP90 (reference band).

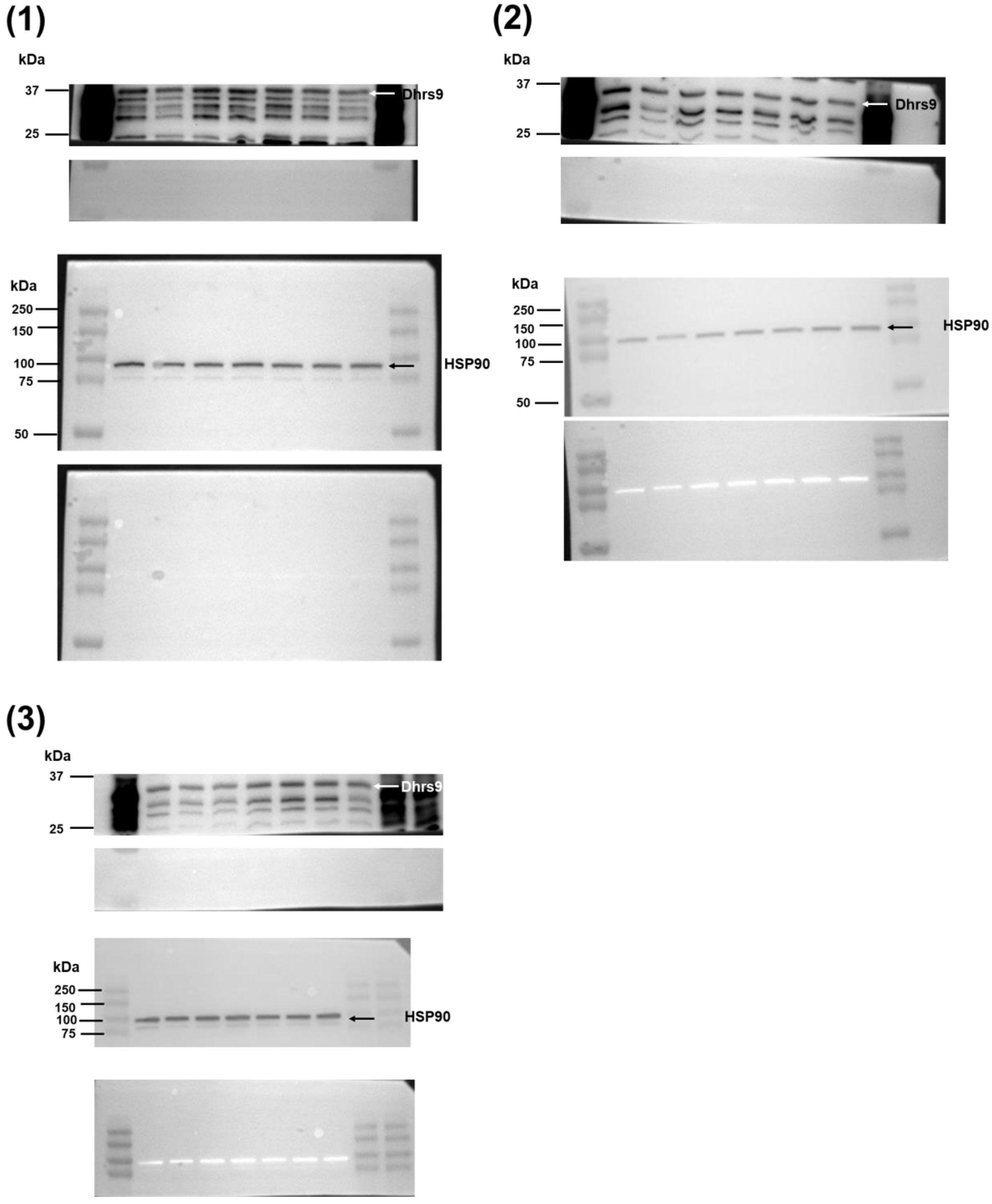

Figure S5. Western blot bands of Dhrs9 and HSP90 (reference band).

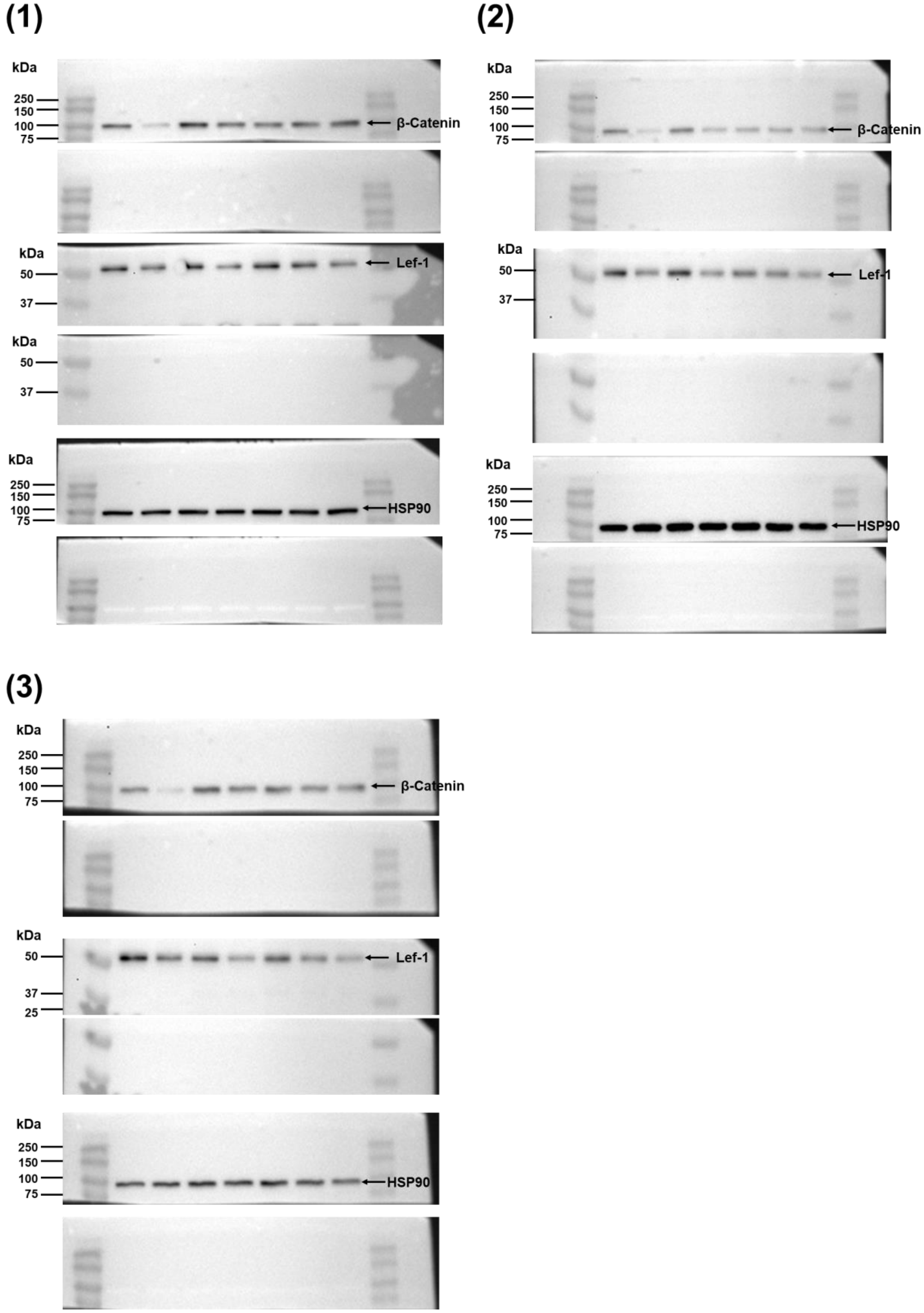

Figure S6. Western blot bands of β-catenin, Lef-1, and HSP90 (reference band).

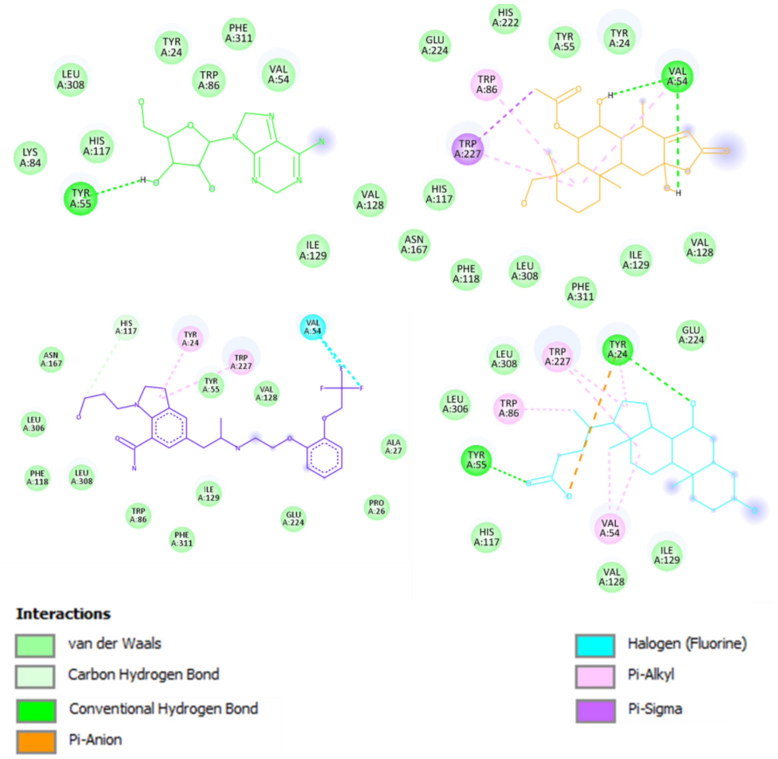

Figure S7. Interactions of adenosine (peak 16), [6,10a-dihydroxy-4-(hydroxymethyl)-4,7,11b-trimethyl-9-oxo-1,2,3,4a,5,6,6a,7,11,11a-decahydronaphtho[2,1-f][1]benzofuran-5-yl] acetate (peak 17), silodosin (peak 57), and redocked native ligand (iso-ursodeoxycholic acid) with key amino acids of Ark1c2 protein (PDB ID: 1IHI).
